# Senescent fibroblast facilitates re-epithelization and collagen deposition in radiation-induced skin injury through IL-33-mediated macrophage polarization

**DOI:** 10.1186/s12967-024-04972-8

**Published:** 2024-02-18

**Authors:** Yan Chen, Le Ma, Zhuo Cheng, Zhihe Hu, Yang Xu, Jie Wu, Yali Dai, Chunmeng Shi

**Affiliations:** Institute of Rocket Force Medicine, State Key Laboratory of Trauma and Chemical Poisoning, Third Military Medical University (Army Medical University), Chongqing, 400038 China

**Keywords:** Senescence, IL-33, Macrophages, Radiation-induced skin injury

## Abstract

**Background:**

The need for radiotherapy among the elderly rises with increasing life expectancy and a corresponding increase of elderly cancer patients. Radiation-induced skin injury is one of the most frequent adverse effects in radiotherapy patients, severely limiting their life quality. Re-epithelialization and collagen deposition have essential roles in the recovery of skin injuries induced by high doses of ionizing radiation. At the same time, radiation-induced senescent cells accumulate in irradiated tissues. However, the effects and mechanisms of senescent cells on re-epithelialization and collagen deposition in radiation-induced skin injury have not been fully elucidated.

**Results:**

Here, we identified a role for a population of senescent cells expressing p16 in promoting re-epithelialization and collagen deposition in radiation-induced skin injury. Targeted ablation of p16^+^ senescent cells or treatment with Senolytics resulted in the disruption of collagen structure and the retardation of epidermal coverage. By analyzing a publicly available single-cell sequencing dataset, we identified fibroblasts as a major contributor to the promotion of re-epithelialization and collagen deposition in senescent cells. Notably, our analysis of publicly available transcriptome sequencing data highlighted IL-33 as a key senescence-associated secretory phenotype produced by senescent fibroblasts. Neutralizing IL-33 significantly impedes the healing process. Finally, we found that the effect of IL-33 was partly due to the modulation of macrophage polarization.

**Conclusions:**

In conclusion, our data suggested that senescent fibroblasts accumulated in radiation-induced skin injury sites participated in wound healing mainly by secreting IL-33. This secretion regulated the local immune microenvironment and macrophage polarization, thus emphasizing the importance of precise regulation of senescent cells in a phased manner.

**Graphical Abstract:**

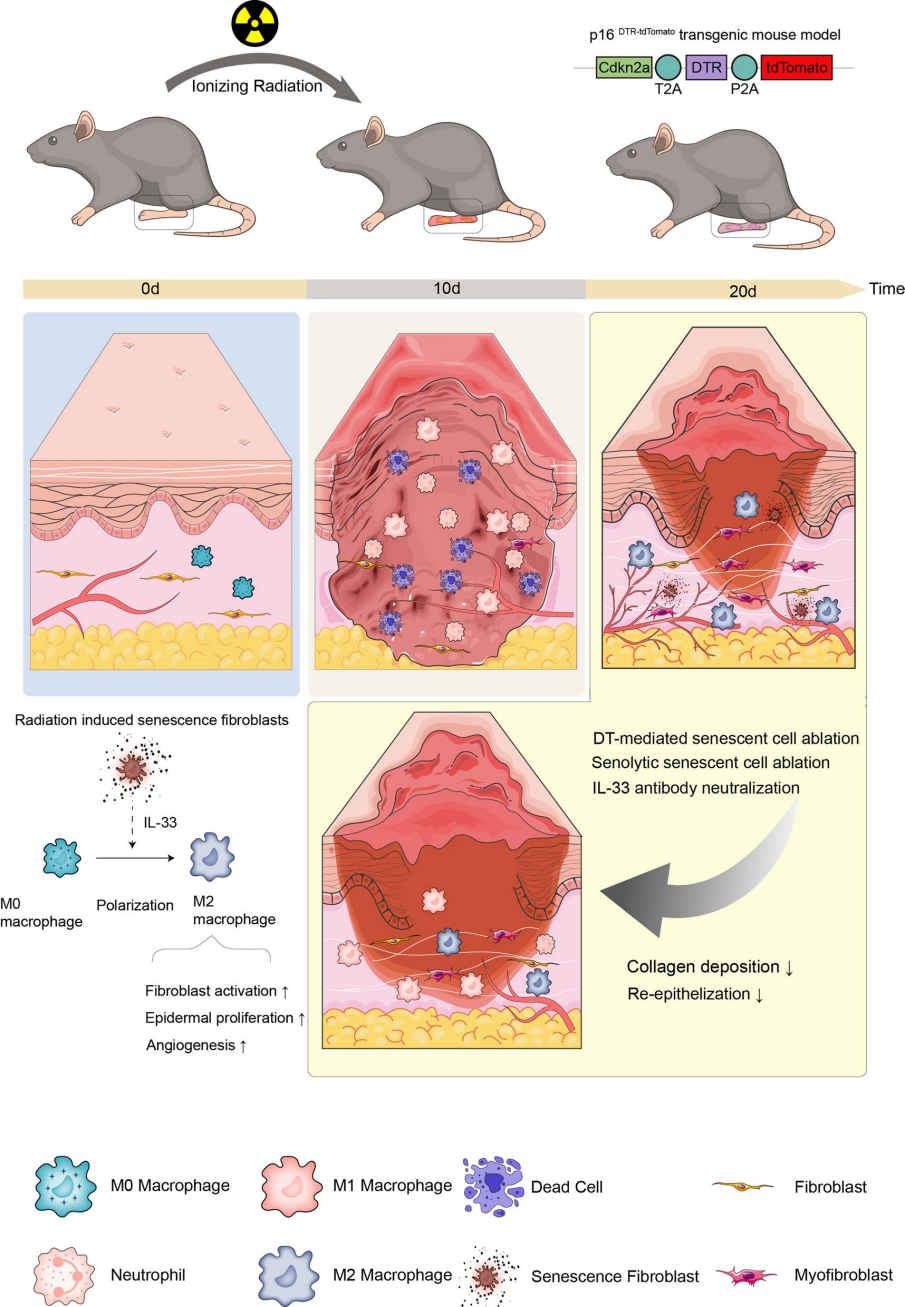

**Supplementary Information:**

The online version contains supplementary material available at 10.1186/s12967-024-04972-8.

## Introduction

Elderly people have a high incidence of tumors, and radiation therapy is commonly used [1]. Approximately 95% of cancer patients receiving radiation therapy are afflicted with radiation-induced skin injury (RISI) [2, 3]. Difficulty in healing severe RISI wounds might be due to radiation-induced alterations in cellular status, which leads to disruption of the healing process. Collagen deposition and epidermal coverage are the core components of skin wound healing and the quality and progression of which directly affect disease prognosis and repair outcomes [4]. However, how radiation-induced changes in the cellular state affect collagen deposition and epidermal coverage is poorly understood. Therefore, it is of great importance to thoroughly investigate the effects and mechanisms of ionizing radiation-induced unique cell states on critical aspects of wound healing and to explore novel intervention strategies accordingly.

Ionizing radiation primarily inflicts significant damage to biomacromolecules, with DNA being particularly susceptible. This results in a series of cellular alterations, including necrosis, apoptosis, and cellular senescence [5–7]. Cells in a senescent state are notable for their enduring tissue presence, resistance to apoptosis, and amplified secretory behaviour [8]. Our previous studies have shown the presence of senescent cells in skin tissue following radiation. Regarding the role of senescent cells in wound repair, some studies have shown that senescent cells are present in cuts, diabetic ulcers, and burn wounds, but their specific role is controversial [9–13]. A possible reason is that senescent cells are not a homogeneous group, and different induction conditions lead to the generation of senescent cells exhibiting different biological functions [14]. However, it is currently unknown whether and how senescent cells are involved in collagen deposition and epidermal coverage in RISI. Our study aimed to investigate the correlation between senescent cells that appear after radiation and key aspects of wound healing in RISI.

Here, we found that a high load of senescent cells was accompanied by vigorous cell proliferation, fibroblast activation, and angiogenesis. By using senolytic drugs or genetic ablation of senescent cells, radiation-induced senescent cells accelerated collagen deposition and re-epithelialization. By scRNA-seq, we further verified that fibroblasts were the primary cell type exerting pro-repair effects in senescent cells. Additionally, we found that IL-33 was one of the most significant cytokines highly expressed by senescent fibroblasts. Mechanistically, IL-33 induced macrophage polarization toward the M2 phenotype, accelerated fibroblast activation, angiogenesis, cell proliferation, and ultimately promoted the re-epithelization and collagen deposition in RISI. These findings provided valuable insights for the development of intervention strategies for RISI with senescent cells as the target.

## Methods and materials

### Animals and treatments

Animal experiments in this study were conducted strictly following protocols approved by the Institutional Animal Care and Use Committee (IACUC) of the Army Medical University. These protocols ensure adherence to ethical guidelines for animal research, prioritizing the welfare and well-being of the animals involved. The C57BL/6J mice used in the study were housed in specific pathogen-free (SPF) animal rooms at a temperature of 23–25 °C and humidity control, with a maximum of 4 mice per cage, kept on a 12-h light/dark cycle (08:00 to 20:00), with standard food and water provided ad libitum to meet their nutritional needs. Environmental enrichments are also provided to ensure physical and mental well-being.

To obtain p16^DTR−tdTomato^ transgenic mice, we performed gene editing using CRISPR‒Cas9 technology. A guide RNA targeting the Cdkn2a gene (TGAGCTAGCTATGCCCGTCGGG), a donor vector containing “T2A-DTR-P2A-tdTomato” and Cas9 mRNA, was coinjected into fertilized eggs of C57BL/6 mice. The F0 generation mice were confirmed by Sanger sequencing. To verify the function of the transgenic mice, we isolated mouse skin fibroblasts (MSFs) from the p16^DTR−tdTomato^ mice and induced cellular senescence by 10 Gy ionizing radiation. As expected, the mRNA levels of Cdkn2a, DTR, and tdTomato were elevated in senescent cells (Additional file 1: Fig. S1A). By immunofluorescence costaining, we confirmed the colocalization of tdTomato fluorescent signals with multiple senescence markers (Additional file 1: Fig. S1B–D). To assess the efficiency of diphtheria toxin in removing senescent cells, we treated senescent MSFs with different concentrations of diphtheria toxin and observed that concentrations equal to or higher than 100 ng/mL of diphtheria toxin showed significant clearance of senescent cells in vitro (Additional file 1: Fig. S1E).

The following drugs were injected intraperitoneally (i.p.): ABT-263 (100 mg/kg; Selleckchem) and diphtheria toxin (25 mg/kg; MilliporeSigma). IL-33 (0.5 μg/mouse; Beyotime) and IL-33 neutralizing antibody (0.5 μg/mouse; R&D Systems) were administered by subcutaneous injection (s.c.). The corresponding solvents were used as controls for all drugs.

For localized ionizing radiation, mice were anesthetized with 4% isoflurane (RWD) and immobilized with a fixator. To protect the surrounding tissues, a 2 cm thick lead sheet was used to shield the area, except for the right leg of the mice. Using an X-ray generator (Precision), 60 Gy of radiation was applied at a 1.3 Gy/min dose rate.

### Histological analysis

For hematoxylin–eosin (HE) staining, sections were deparaffinizationed with xylene and gradient ethanol and immersed in hematoxylin staining solution for 2 min, rinsed with water, and differentiated with hydrochloric acid–ethanol. Subsequently, the sections were briefly immersed in eosin staining solution, dehydrated in gradient ethanol, and finally sealed with a drop of neutral gum after 10 min of transparency in xylene.

For Masson’s trichrome staining, sections were immersed in Boulin’s fixative overnight and then treated sequentially with azurite blue for 3 min, Mayer hematoxylin for 3 min, hydrochloric acid‒alcohol fractionation solution for 2 s, Reichhorn red for 10 min, phosphomolybdate for 10 min, and aniline blue for 5 min. Sections were dehydrated with graded ethanol, cleared with xylene for 10 min, and finally sealed with a drop of neutral gum.

For immunofluorescence and immunohistochemical staining, tissue sections were deparaffinized and repaired with citrate for antigen repair, blocked with goat serum, and incubated with the primary antibody at 4 °C overnight. After washing with TBST, the sections were incubated with secondary antibody at room temperature for 2 h. DAPI or hematoxylin was used for nuclear staining. Anti-tdTomato (1:200; OB-RB013-02, Oasis) was used to detect tdTomato expression with a low fluorescence signal. Images were acquired using an Olympus VS200 microscope. ImageJ software was used to measure the percentage of positive cells relative to the DAPI signal or the percentage of positive signal area in each sample’s 12 high magnification fields of view. Negative controls were performed using the same specimens without adding primary antibodies. The primary antibodies used in the study were ki-67 (1:200; ab15580, Abcam), α-SMA (1:200; A2547, Millipore), CD31 (1:50; ab28364, Abcam), and P16 (1:200; ab54210, Abcam).

For TdT-mediated dUTP Nick-End Labeling (TUNEL) staining, the Roche In Situ Cell Death Detection Kit, POD, 11,684,817,910 was used. Sections were dewaxed, and treated with Proteinase K for 30 min at room temperature, washed, and TUNEL reaction mix (containing 50 μl TdT and 450 μl fluorescein-labeled dUTP solution) was added dropwise. The negative control group was treated with 450 μl fluorescein-labeled dUTP solution only, the positive control group was treated with 100 μl DNase1 for ten minutes after proteinase K treatment, and the rest of the steps were the same), incubated for 1 h at room temperature protected from light, TBST washed. DAPI-containing blocking solution (C1002, Beyotime) was added dropwise and covered with a coverslip, and the sections were incubated using the Invitrogen EVOS fluorescence microscope for image acquisition. ImageJ software was used for quantitative analysis.

### Tissue dissociation and flow cytometry analysis

Mouse skin samples were minced directly using a sharp blade and then incubated in Dulbecco’s modified Eagle’s medium (DMEM) containing 1 mg/mL collagenase IV (9001–12-1, Gibco) and 12.5 kU DNase (D4263, Sigma) at 37 °C for 90 min. Following incubation, the cells were filtered through a 70 μm cell strainer. Subsequently, the cells were treated with an FcR blocking reagent (156604, Biolegend) at 4 °C for 10 min and then stained with fluorescence-labeled antibodies against CD45 (157214, Biolegend), CD26 (45-0261-82, eBioscience), CD44 (17-0441-82, eBioscience), F4/80 (123131, Biolegend), CD31 (63-0311-82, eBioscience), or isotype control at 4 °C for 30 min. After washing, the samples were analyzed using FACS Aria II (BD Biosciences).

### Fluorescence-activated cell sorting of senescent and non-senescent fibroblasts

After euthanasia, the skin of irradiated mice was shredded using a sharp blade. Subsequently, the skin was incubated in DMEM containing 0.5 mg/mL collagenase I (A004194, Diamond), 0.5 mg/mL collagenase II (A004174, Diamond), 1 mg/mL collagenase IV (A004186, Diamond), 0.5 mg/mL hyaluronidase (H3506, Biosharp), 0.5 mg/mL elastase (A002290, Diamond) and 12.5 kU DNase (D4263, Sigma). Incubation was performed at 37 °C for 90 min. After incubation, cells were filtered through a 70 μm cell strainer. Subsequently, cells were treated with FcR blocking reagent (156604, Biolegend) for 10 min at 4 °C, then stained with fluorescently labeled CD26 antibody, and dead cells were filtered using DAPI. Senescent cellular fibroblasts with DAPI^**−**^ CD26^**+**^ tdTomato^**+**^ and non-senescent fibroblasts with DAPI^**−**^ CD26^**+**^ tdTomato^**−**^ were isolated using a BD FACS Aria II, and culture was continued in DMEM.

### Primary cell isolation and culture

Primary mouse keratinocytes were extracted using the human whole skin dissociation kit from Miltenyi Biotec and fluorescence-activated cell sorting (FACS). The procedure involved peeling off the skin from the entire leg using sterile surgical instruments and carefully removing subcutaneous fascia and adipose tissue. The skin was then immersed in a penicillin–streptomycin antibiotic solution for 10 min to maintain sterility. Next, the digestive enzyme complex from the skin dissociation kit was added to the Miltenyi C tube, and the tube was incubated at 37 °C for 3 h. After incubation, 3 mL of DMEM culture medium was added to the digested suspension. The suspension was then mixed with a mild MACS dissociator from Miltenyi Biotec and the C-tube to obtain a single-cell suspension. CD45^−^ and CD44^+^ cells were isolated using a BD FACS Aria II and cultured in DMEM to isolate keratinocytes from single-cell suspensions.

For BMDM extraction, bone marrow cells were extracted from normal mouse femurs and cultured in RPMI 1640 medium with 10% fetal bovine serum and 20 ng/mL macrophage colony-stimulating factor (Beyotime) for 6 days. Subsequently, BMDMs were stimulated with 100 ng/mL lipopolysaccharide (Sigma‒Aldrich), 10 ng/ml interleukin-13 (Beyotime), and 20 ng/mL interleukin-33 (Beyotime) for 48 h. After stimulation, the percentages of CD86^+^ (105008, Biolegend) and CD206^+^ (321109, Biolegend) cells were analyzed using an Attune flow cytometer (Thermo).

### EdU cell proliferation assay

To perform EdU experiments, we incubated primary keratinocytes or fibroblasts in different conditioned media for 48 h. Subsequently, EdU was added to the medium to achieve a final concentration of 10 μM. After 2 h of incubation, cells were collected after trypsin digestion, fixed with paraformaldehyde, and the membrane was broken with Triton X-100. Then, a reaction solution containing click reaction buffer, CuSO4, Azide 555, and click additive solution was introduced into the cell culture medium, and the cells were incubated in the dark for 30 min. Finally, the cells were rinsed three times with PBS. The percentage of EdU^+^ signal was analyzed using a BD Accuri C6 Plus flow cytometer.

### Transwell and scratch assays

Cell migration was assayed using in vitro cell scratch and Transwell migration assays. Conditioned medium was added to the lower chamber of the Transwell, and primary keratinocytes or fibroblasts were inoculated into the upper chamber of the Transwell. After overnight incubation, the upper chamber was removed, and the migrated cells were fixed in 4% paraformaldehyde for 15 min. After fixation, the cells were stained with crystal violet solution for 30 min. Subsequently, the chambers were washed with PBS to remove excess staining. The microtiter membrane containing migrated cells was then carefully cut off and placed on a slide. Images were acquired using an Invitrogen EVOS microscope. To quantify the extent of cell migration, migrating cells were counted in 12 high-magnification fields of view using ImageJ software.

For the scratch assay, primary keratinocytes or fibroblasts were cultured in 6-well plates and incubated until reaching 80% confluence the day before the experiment. Before the experiment, a consistent scratch was created at the bottom of the well using a pipette tip, followed by washing with PBS. Next, the cells were cultured for 24 h, after which the culture medium was removed, and the cells were fixed with 4% paraformaldehyde for 15 min to capture the subsequent image. Brightfield images were acquired using an Invitrogen EVOS microscope, and quantitative analysis of the scratch area was performed using ImageJ software.

### Quantitative RT‒PCR

Total RNA was extracted from samples using TRIzol reagent (Thermo). Subsequently, 1 μg of RNA was reverse transcribed into cDNA following the recommended protocol of the RevertAid First Strand cDNA Synthesis kit (Thermo). Quantitative RT‒PCR was conducted according to the recommended protocol using SYBR Green qPCR master mix (Takara). The relative gene expression was calculated using the comparative threshold cycle (Ct) method, with ACTB expression as the loading control. The primer sequences for mouse-specific genes are listed in Additional file 1: Table S2.

### Western blot analysis

Total protein was extracted from mouse skin tissues using ice-cold RIPA lysis buffer (Beyotime) containing a protease inhibitor mixture (Roche). The total protein content was quantified using the BCA method. Protein samples were subjected to Mini-PROTEAN^®^ TGX Stain-Free™ Precast 10% or 12% gels (Bio-Rad) and transferred to 0.2 µm PVDF membranes (Millipore) using the Trans-Blot Turbo Transfer Pack and Trans-Blot Turbo Transfer System (Bio-Rad). PVDF membranes were incubated with primary antibody overnight at 4°C, followed by HRP-coupled secondary antibody for 1 h at room temperature. Bio-Rad ChemDoc MP was used for Western blot data collection and analysis. The primary antibodies used in this study were Tgf-β (1:1000; ab215715, Abcam), IL-6 (1:1000; ab9324, Abcam), γ-H2AX (1:1000; ab81299, Abcam) and p16 (1:1000; sc1661, Santa Cruz).

### scRNA-seq data analysis

The downstream analysis of scRNA-seq data was made possible by the single-cell toolkit Seurat (version 5.0.1) [15] in R (version 4.3.0). To ensure high-quality cells, we only included cells with more than 500 and less than 5000 detected genes and less than 20% mitochondrial genes for subsequent analysis. Additionally, we used the R package DoubletFinder (version 2.0.3) [16] to identify and remove doublets from the scRNA-seq data in each sample. To reduce biologically heterogeneous false positives in scRNA-seq data caused by technical factors such as sequencing depth, we normalized and scaled the data using the R package SCTransform (version 0.4.1) [17]. This allowed for clearer identification of biological differences. We used the ‘PrepSCTIntegration’ and ‘FindIntegrationAnchors’ functions to select integration anchors and perform downstream integration. The dataset of all samples is integrated using the ‘IntegrateData’ function with the anchors. The integrated dataset is then utilized for downstream downscaling and cluster analysis. Cell clustering was performed using the ‘FindClusters’ function at a resolution of 2.0, and the first 15 principal components (PCs) were used to define cell identity. Size reduction was performed using the 'RuntSNE' function. Marker genes for each cluster were identified using the Wilcoxon rank sum test through the ‘FindAllMarkers’ function. Only genes with an ‘avg_logFC’ greater than 0.5 and a ‘p_val_adj’ less than 0.05 were considered as marker genes. Cell types were identified based on the expression of classical marker genes. To identify senescent cells, we scored the Reactome cell senescence gene set [18] using the ‘AddModuleScore’ function. Cells with senescence scores above the mean plus 2 standard deviations were categorized as senescent cells. For gene set enrichment analysis (GSEA), after obtaining senescent and non-senescent cell differential genes in different cell types by using the ‘FindAllMarkers’ function, the genes were sorted using Wald statistics using the gseaplot2 function in the R package clusterProfiler (version 4.8.3) [19] to calculate the normalized enrichment score (NES) and p-value.

### Bulk RNA-seq data analysis

The number of gene counts was first calculated using the control GRCh38, GRCm39, or mRatBN7.2 reference genomes using HISAT2 (version 2.2.1) [20]. For gene expression quantification using HTSeq (version 2.0.5) [21], only high-quality calibration reads (calibration quality score > 20) were counted. Differential expression analysis was performed with DESeq2 (version 3.1.8) [22]. Differentially expressed genes (DEGs) in the irradiated and control groups were identified using Benjamini Hochberg (BH) adjusted P-values < 0.05 and |log2FC| > 1 as cutoffs.

### Statistical analysis

Statistical analysis was conducted using R 4.2.1 software, and the results are presented as the mean ± standard deviation (SD). The *t*-test was applied to compare differences between two groups with equal variances, and one-way analysis of variance (ANOVA) was used to compare differences between three or more groups. In the case of unequal variances, the Mann‒Whitney *U* test was used. A p-value of less than 0.05 was considered statistically significant, indicating a significant difference between the compared groups.

## Results

### The accumulation of senescent cells in RISI is accompanied by collagen deposition, epidermal proliferation, and angiogenesis

**Fig. 1:**
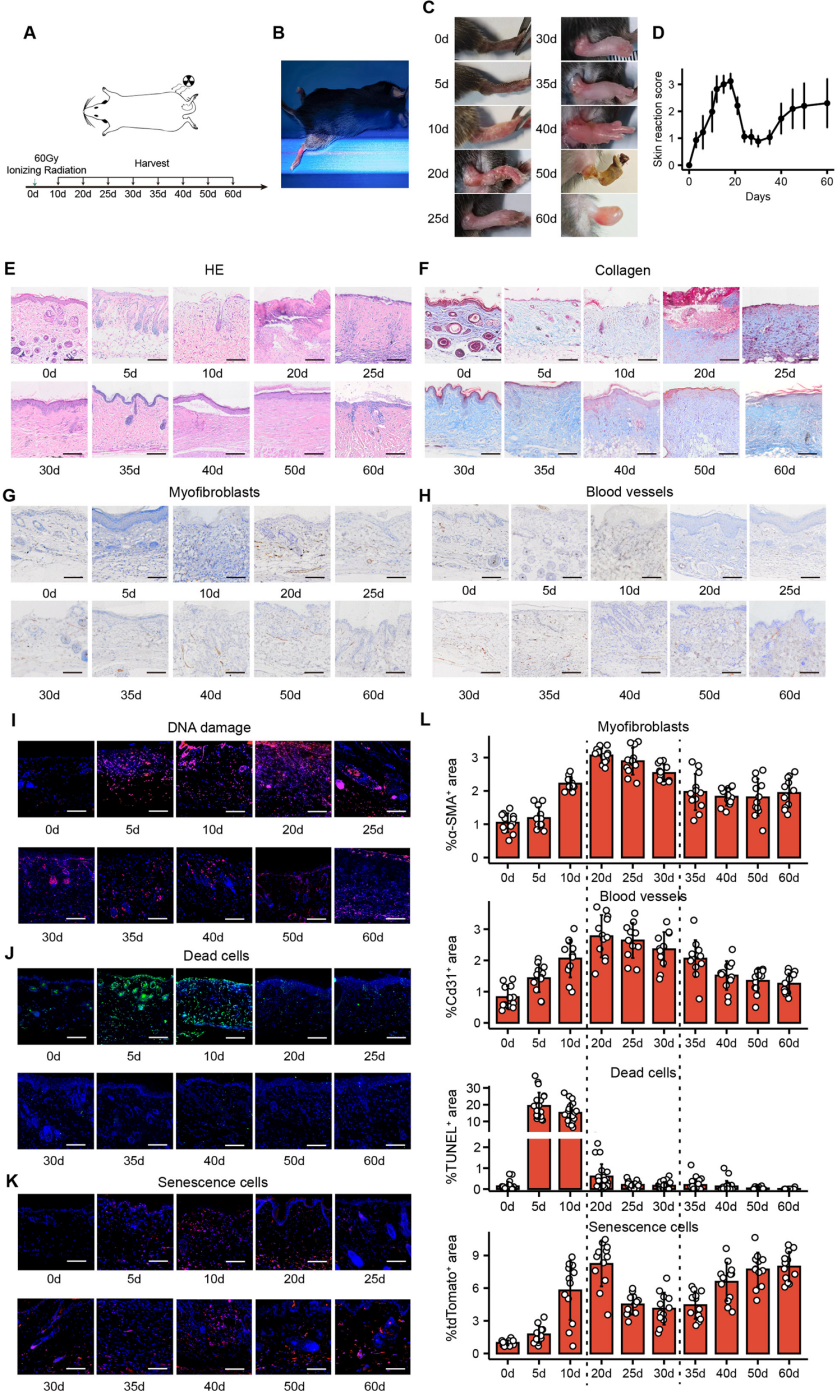
Fibroblast activation, cell proliferation, and vascular infiltration accompany the appearance of senescent cells. **A** Establishment of the mouse RISI model and schematic diagram of sample collection. **B** Schematic illustration of the irradiated area in the RISI model. **C** Representative images of the general appearance of mice at different time points after irradiation. **D** Gross injury score, with scoring ranging from 0 (no injury) to 3.5 (complete moist breakdown of limb—often stuck to body); n = 5 for each group. Representative images of **E** HE staining, **F** Masson staining, **G** α-SMA^+^ myofibroblasts and **H** CD31^+^ endothelial cells at different times post-irradiation. Representative images of **I** γ-H2AX^+^ DNA damage cells, **J** TUNEL^+^ dead cells and **K** p16^+^ senescent cells immunohistochemical staining at various times post-irradiation. **L** Quantitative analysis of the positive signals of α-SMA, CD31, TUNEL, and p16 at different times after irradiation. 12 high-magnification fields were randomly selected, and the positive signal area was analyzed using ImageJ software. Data were presented as mean ± standard deviation. γ-H2AX standed for Phosphorylated histone H2AX, DAPI for 4′,6-diamidino-2-phenylindole, and TUNEL for terminal deoxynucleotidyl transferase-mediated fluorescein-dUTP nick-end labeling. Bars represented 100 μm

To investigate the relevance of radiation-induced changes in cellular status in the process of RISI, we developed a model of severe RISI induced by high-dose ionizing radiation and characterized its histopathology. Using the irradiation protocol delineated by IWAKAWA [23] and building on the foundational findings of previous studies [24–26], we delivered a singular dose of 60 Gy to the right hind limbs of mice at a rate of 1.3 Gy/min. The sampling methodology is shown in Fig. 1A, while the irradiation area on the mice is illustrated in Fig. 1B. Based on the criteria in Additional file 1: Table S1, we attributed relevant scores. The severity of the injury was gauged, and scores between 0 and 3.5 were allocated.

Mouse skin showed typical injury features after irradiation, including erythema, edema, desquamation, ulceration, and fibrosis (Fig. 1C, D). HE and Masson staining showed pathological changes similar to those observed in the macrosomic samples (Fig. 1E, F). Epidermal exfoliation and ulcer formation occurred predominantly between 10 to 20 days, and proliferative responses, such as epidermal coverage and collagen production, occurred predominantly between 20 to 30 days. Subsequently, we evaluated the levels of fibroblast activation and vascular infiltration in the proliferative response. α-SMA and CD31 immunohistochemistry showed a significant increase in the expression of both from days 20 to 30 after irradiation, followed by a gradual decrease (Fig. 1G, H). The results showed that the destruction of tissue structure and the formation of ulcers began 10 days after radiation, and the peak of the proliferative response, such as epidermal hyperplasia, collagen deposition, and vascular infiltration, was mainly concentrated in the period of 20 to 30 days after radiation.

To study radiation-specific cellular events during tissue repair, we first assessed the extent of DNA damage after radiation. Utilizing immunofluorescence staining of the DNA damage marker γ-H2AX, we identified enduring DNA damage after irradiation that gradually decreased over time (Fig. 1I). Typically, cells respond to DNA damage in three ways: recovery, senescence, and death [27]. To measure the temporal distribution of cell death in RISI, we used the TUNEL staining technique. The results showed that a large number of dead cells distributed in the epidermis and dermis appeared within the first 10 days after irradiation, and the number decreased after 20 days (Fig. 1J, L). This coincided with the time of epidermal exfoliation and ulcer formation, verifying that the loss of tissue integrity induced by cell death was the direct cause of ulcer formation or wet desquamation [28]. Cellular senescence was another consequence of DNA damage. p16 exerts a function in limiting cell cycle progression and is considered a potent marker of senescent cells. p16 expression is weak in healthy cells, but in senescent cells, p16 binds to and inhibits cell cycle protein-dependent kinase 4/6 (CDK4/6) activity, thereby promoting retinoblastoma (RB)-dependent cell cycle arrest [29–31]. By detecting the senescent cell marker p16 using immunofluorescence staining, it was found that senescent cells remained present for extended periods following irradiation. In addition, a significant increase in the number of senescent cells was observed at the peak of re-epithelialization, collagen deposition, and angiogenesis (Fig. 1K, L). Western blot analysis further confirmed the persistence of DNA damage and cellular senescence after radiation (Additional file 1: Fig. S2).

### Pharmacogenetic ablation of p16^+^ cells or senolytic treatment delays fibroblast activation and epidermal proliferation

**Fig. 2:**
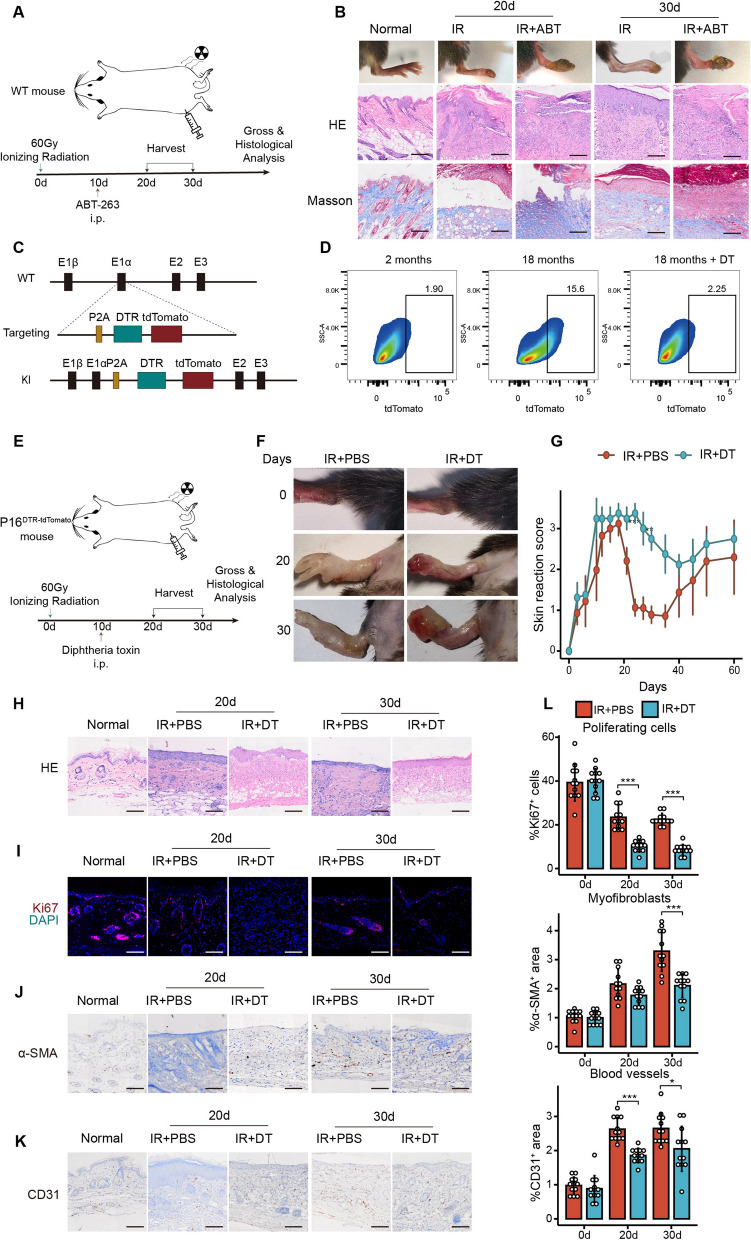
Senescent cell intervention impacts re-epithelialization and collagen deposition. **A** Schematic diagram of senolytic ABT-263 clearing senescent cells and sample collection. **B** Representative gross images, HE staining images, and Masson staining images showing reduced damage in the irradiated limbs after ABT-263 treatment compared to the PBS control, n = 5. **C** Schematic illustration of the target construction for p16^DTR−tdTomato^ mice and CRISPR gene editing. **D** Representative data from flow cytometry analysis of the proportion of tdTomato^+^ in single-cell suspensions of skin from young mice (2 months old), aged mice (18 months old), and aged mice after senescent cells clearance, n = 5. **E** Schematic diagram of using diphtheria toxin to target and clear senescent cells and sample collection. **F** Representative gross images showing reduced damage in the irradiated limbs after diphtheria toxin treatment compared to the PBS control. **G** Gross injury scores comparing the senescent cells clearance groups and the control groups, n = 3 for each group. Representative images of **H** HE staining, **I** Ki67^+^ proliferative cells immunofluorescent staining, **J** α-SMA^+^ myofibroblasts immunohistochemical staining and CD31^+^ **K** endothelial cells immunohistochemical staining comparing the senescent cells clearance groups and the control groups. **L** Quantitative analysis of the positive signals of Ki67, α-SMA, and CD31 between the senescent cell clearance groups and the control groups. 12 high-magnification fields were randomly selected, and the proportion of positive signal cells (Ki67) or the area of positive signals (α-SMA, CD31) were analyzed using ImageJ software. Data were presented as mean ± standard deviation. When variance was met, a *t*-test was used for statistical analysis between two groups, while ANOVA was used for comparisons among three or more groups. If variance wasn’t met, the Mann-Whitney *U* test was used. *p < 0.05, **p < 0.01, ***p < 0.001. Bars represented 100 μm

Given the previous finding that senescent cells were predominantly clustered in the peak of re-epithelialization, collagen deposition and angiogenesis of RISI, our next aim was to assess the biological effects of senescent cells. For this purpose, we utilized ABT-263, a renowned senolytic agent proven to effectively target and eliminate senescent cells, as corroborated by multiple studies [32–36]. We injected ABT-263 into our samples on day 10 following irradiation to eradicate senescent cells, and assessed the macroscopic and histopathological changes (Fig. 2A) subsequently. Mice that underwent treatment with a senolytic agent exhibited poorer healing outcomes, including premature epidermal detachment, exacerbation of wet exudates and ulcers, increased hemorrhage, and a delayed recovery rate compared to controls. HE staining demonstrated accelerated epidermal exfoliation and enhanced inflammatory infiltration after senescent cell removal, accompanied by delayed angiogenesis and epidermal coverage. Furthermore, Masson staining revealed a delay in collagen deposition (Fig. 2B).

Considering the possibilities of ABT-263’s off-target effects and incomplete clearance of senescent cells[33], we sought to provide a more comprehensive evaluation of the distribution and functionality of these cells. Our method involved the development of transgenic mice equipped to both track and eliminate senescent cells. Utilizing CRISPR‒Cas9 technology, we successfully incorporated both the diphtheria toxin receptor and the fluorescent protein tdTomato downstream of the established senescent cell marker CDKN2A. This integration allowed for the visualization of senescent cells through fluorescence and facilitated their removal using diphtheria toxin (DT) (Fig. 2C). To verify the ability of p16^DTR−tdTomato^ mice to track and remove senescent cells in vivo, we obtained single-cell suspensions of the skin from aged transgenic mice treated with diphtheria toxin and analyzed with flow cytometry and immunofluorescence (Fig. 2D, Additional file 1: Fig. S3A). The results showed that senescent tdTomato^+^ cells significantly accumulated in the skin of aged mice, and diphtheria toxin administration significantly reduced the abundance of senescent cells. We then applied this model to inspect the distribution of senescent cells after irradiation. Consistent with previous p16 immunofluorescence staining results, the transgenic model showed high expression of tdTomato during the tissue repair phase of RISI, which was further validated by flow cytometry and tdTomato fluorescence (Additional file 1: Fig. S3B, S3C).

To further investigate the role of senescent cells in the repair of RISI, we targeted these cells for elimination using diphtheria toxin 10 days post-irradiation and subsequently evaluated their biological functions. The genetic strategy deployed for the clearance of senescent cells is illustrated in Fig. 2E. We consistently assessed and scored the overall condition of the irradiated limbs. Representative macroscopic images showed more severe epidermal detachment, inflammatory exudation, and hemorrhage in the limbs of the irradiated mice after the removal of senescent cells. These observations were supported by markedly lower scores than those of the control group (Fig. 2F, G). HE staining (Fig. 2H) showed that removing senescent cells resulted in a more severe loss of subcutaneous appendages accompanied by marked epidermal detachment, necrosis, and disruption of dermal fiber structure. After the removal of senescent cells, the percentage of Ki67^+^ cells, a marker of cell proliferation, was significantly reduced, especially in the basal layer of the epidermis (Fig. 2I, L). In addition, the level of α-SMA, a fibroblast marker indicative of collagen deposition, and CD31, an endothelial cell marker indicative of vascular infiltration, were also significantly reduced after the removal of senescent cells (Fig. 2J–L). It has been shown that radiation-induced senescent cells promote keratinocyte proliferation, fibroblast activation, and angiogenesis to enhance epithelial reformation and collagen deposition in RISI.

### Fibroblasts are the central subpopulation of irradiated senescent cells that promote re-epithelialization and collagen deposition

**Fig. 3:**
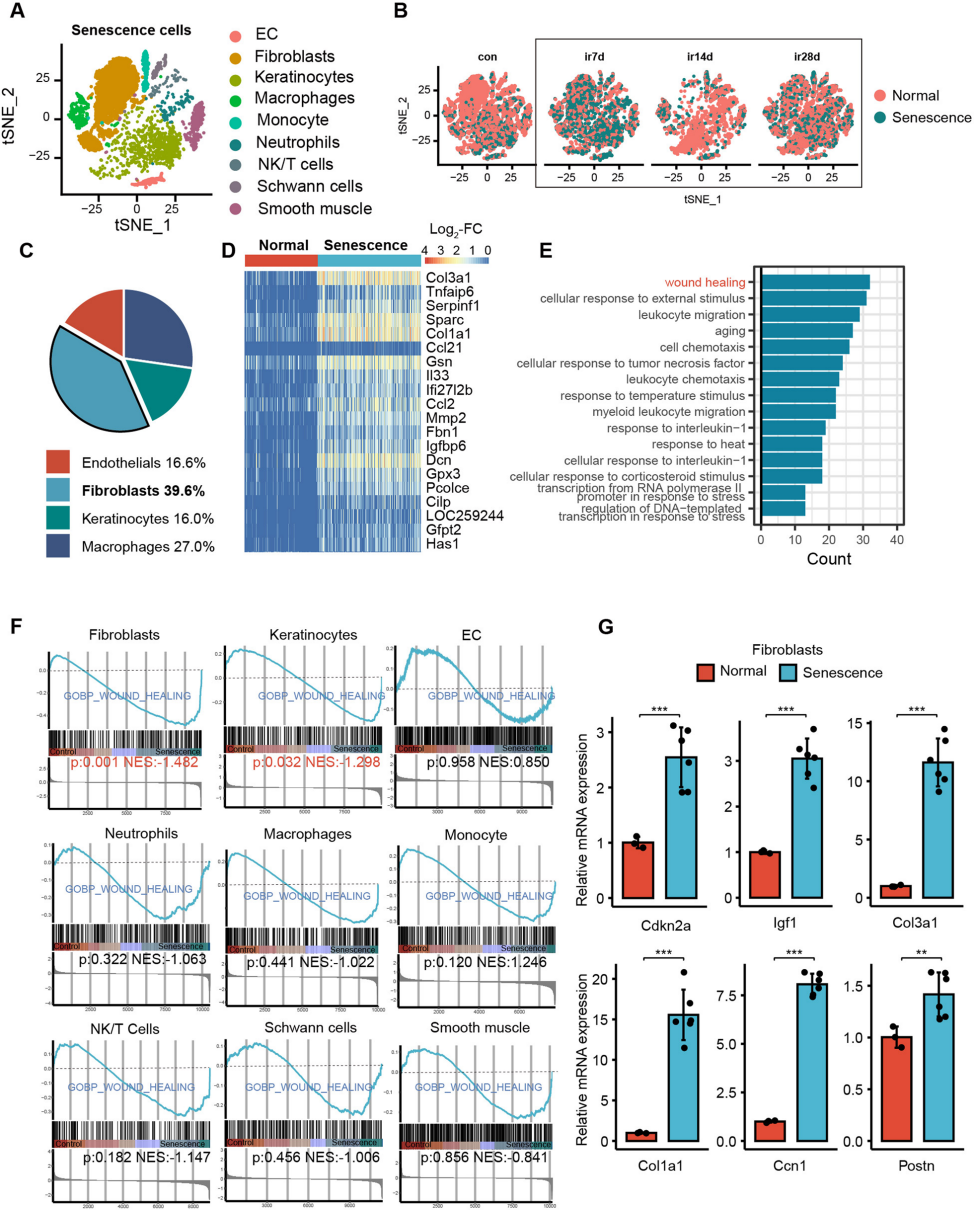
Senescent fibroblasts are the main contributors to the healing of RISI. **A** t-SNE dimensionality reduction showed the cell types identified in the single-cell dataset. **B** Display of senescent cells at different time points post-irradiation in the single-cell dataset. **C** Flow cytometry sorts and displays the proportional distribution of various types of senescent cells, with fibroblasts being the predominant type. **D** Heatmap demonstrates the representative differentially expressed genes that were highly expressed in senescent cells. **E** GO analysis indicated the enrichment of wound healing pathways in senescent cells. **F** GSEA compares the enrichment of wound healing pathways among different subtypes of senescent cells; fibroblasts and keratinocytes showed high expression of this pathway. **G** qPCR analysis of up-regulated wound healing-related genes in senescent fibroblasts sorted 20 days after irradiation, compared to the control group, n = 6. When variance is met, a *t*-test was used for statistical analysis between two groups, while ANOVA was used for comparisons among three or more groups. If variance wasn’t met, the Mann-Whitney *U* test was used. *p < 0.05, **p < 0.01, ***p < 0.001

Given the function of senescent cells in promoting re-epithelialization and collagen deposition, our study aimed to further explore the cell types that exert a major role. For this purpose, we analyzed the single-cell transcriptome sequencing dataset submitted by Tu et al. [37] (GSE193564). This dataset involved exposing rat skin to 40 Gy of ionizing radiation, and irradiated and non-irradiated skin samples were collected at various time points. Following dissociation, single-cell transcriptome sequencing was performed, and the Seurat package was employed for unsupervised clustering analysis and dimensionality reduction, leading to the identification of nine main cell types (Fig. 3A).

By using cellular senescence markers, we identified and screened senescent cells in the scRNA-seq dataset (Fig. 3B). Senescent cells were distributed in a variety of cell types. Among them, fibroblasts, macrophages, endothelial cells, and keratinocytes accounted for the highest proportion of senescent cells. We subsequently extracted single-cell suspensions from samples 10 days post-radiation and analyzed using flow cytometry, which showed that fibroblasts dominated in the number of senescent cells (Fig. 3C). By analyzing the differentially expressed genes in senescent cells versus control normal cells and shown by heatmap, we observed significant differences in the gene expression profiles of the two groups (Fig. 3D). Gene Ontology (GO) enrichment analysis showed that senescent cells were significantly enriched in the “wound healing” pathway during the repair phase, further confirming the involvement of senescent cells in tissue repair of RISI (Fig. 3E).

Next, we analyzed nine cell types by GSEA to find the senescent cell type with the most significant impact on wound healing. The results showed differences in the NES of senescent fibroblasts and keratinocytes, with fibroblasts scoring the highest. This finding suggested that fibroblasts may be the main cell type that exerts pro-repair effects in senescent cells (Fig. 3F). Subsequently, senescent and non-senescent fibroblasts were separated using FACS. The five most highly expressed genes in the "GOBP_WOUND_HEALING" pathway in senescent fibroblasts from scRNA-seq and the senescence marker Cdkn2a were verified by qPCR. The results demonstrated elevated levels of p16 in the sorted senescent cells, along with upregulation of wound healing-related genes (Fig. 3G). These results highlighted the importance of senescent fibroblasts in enhancing re-epithelialization and collagen deposition. Therefore, we conducted further studies on senescent fibroblasts.

### IL-33 is the key senescence-associated secretory phenotype secreted by senescent fibroblasts

**Fig. 4:**
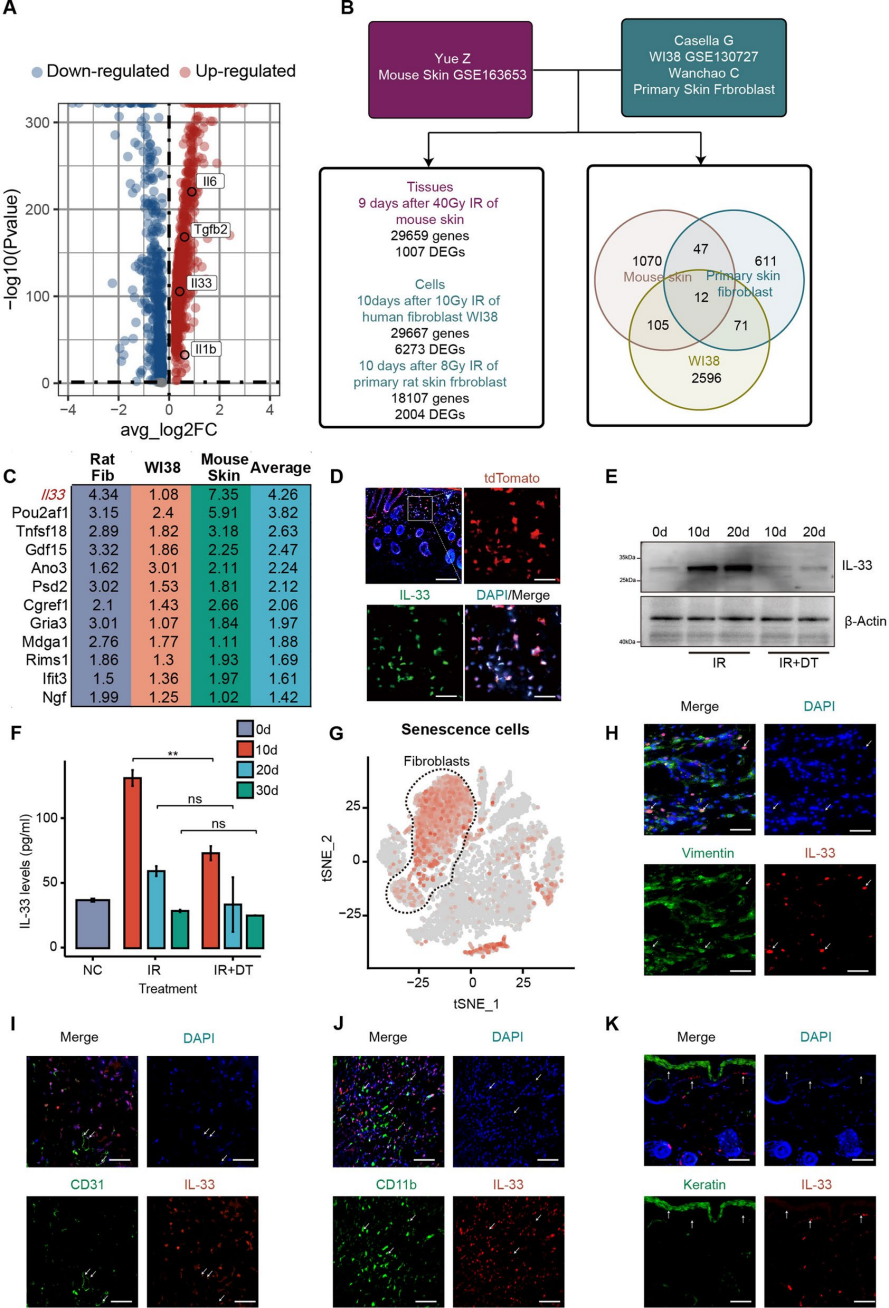
IL-33 serves as the primary SASP secreted by senescent fibroblasts. **A** Volcano plot of differentially expressed genes between senescent fibroblasts and control group in the scRNA-seq dataset, showing representative SASP expression levels. **B** Schematic of integrated transcriptomic analysis, including gene sets and Venn analysis results. **C** Expression levels of commonly highly expressed genes across different gene sets, in Log_2_FC units, with IL-33 being the most highly expressed gene. **D** Representative immunofluorescence co-staining experiments show that IL-33 was primarily co-localized with senescent cells, n = 3. **E** Western blot revealed a significant reduction in IL-33 expression levels after the removal of senescent cells, with the experiment repeated three times, and a representative image was shown. **F** Elisa results indicated that serum IL-33 secretion levels significantly decreased after senescent cells removal, n = 3. **G** t-SNE dimensionality reduction image displayed that IL-33 was primarily expressed in fibroblasts. **H**–**K** Representative immunofluorescence co-localization images of IL-33 in fibroblasts, vascular endothelial cells, and myeloid immune cells, showing that IL-33 was mainly expressed in fibroblasts. Arrows point to IL-33^+^ cells, with the experiment repeated three times. When variance was met, a *t*-test was used for statistical analysis between two groups, while ANOVA was used for comparisons among three or more groups. If variance wasn’t met, the Mann-Whitney *U* test was used. *p < 0.05, **p < 0.01, ***p < 0.001. Bars represented 100 μm (**D**), 50 μm (**H**–**K**)

As we know, senescent cells exhibit heightened metabolic activity and express the senescence-associated secretory phenotype (SASP) due to cell cycle arrest, which is the primary mechanism which they exert their biological effects [38]. To investigate the effector molecules of senescent fibroblasts that promote re-epithelialization and collagen deposition, we screened their expression of SASP. By analyzing DEGs in senescent fibroblasts using scRNA-seq analysis, we discovered that senescent fibroblasts exhibited elevated expression of multiple repair-promoting factors, including IL-33 and TGF-β (Fig. 4A).

Subsequently, we conducted a bulk RNA-seq joint analysis of skin tissue and fibroblast datasets related to RISI from the GEO database. As depicting in Fig. 4B, we integrated three datasets, including a human diploid fibroblast cell line WI38 dataset (10 Gy, 10 days post-irradiation, GSE130727), a mouse skin dataset (40 Gy, 9 days post-irradiation, GSE163653), and our previously obtained dataset of primary rat skin fibroblasts (8 Gy, 10 days post-irradiation) [39]. Venn analysis of DEGs across these three datasets revealed 12 genes with consistently elevated expression. Among these, IL-33 showed the most significant increase (Fig. 4C), suggesting that IL-33 may represent a stable and significant SASP factor expressed by radiation-induced senescent fibroblasts.

To validate the relationship between IL-33 and senescent cells, we verified the co-localization of IL-33 with senescent cells by immunofluorescence (Fig. 4D). Further, Enzyme-linked immunosorbent assay (ELISA) and Western blot demonstrated that clearance of senescent cells after irradiation significantly decreased IL-33 levels (Fig. 4E, F). These findings provide compelling evidence that the expression of IL-33 is mainly derived from senescent cells. Subsequently, we identified the predominant cells expressing IL-33 by single-cell sequencing. Our research findings revealed that senescent endothelial cells and fibroblasts displayed the highest levels of IL-33 expression (Fig. 4G). Subsequently, immunofluorescence co-localization experiments in multiple cell types revealed that most IL-33 fluorescent signals originated from fibroblasts, a few from vascular endothelial cells and keratinocytes, and almost no IL-33 expression in myeloid immune cells (Fig. 4H–K). These results suggested that senescent fibroblasts stably express IL-33 as SASP, which may contribute to the improvement of re-epithelialization and collagen deposition effects.

### IL-33 facilitates the repair of RISI and alters the immune microenvironment

**Fig. 5:**
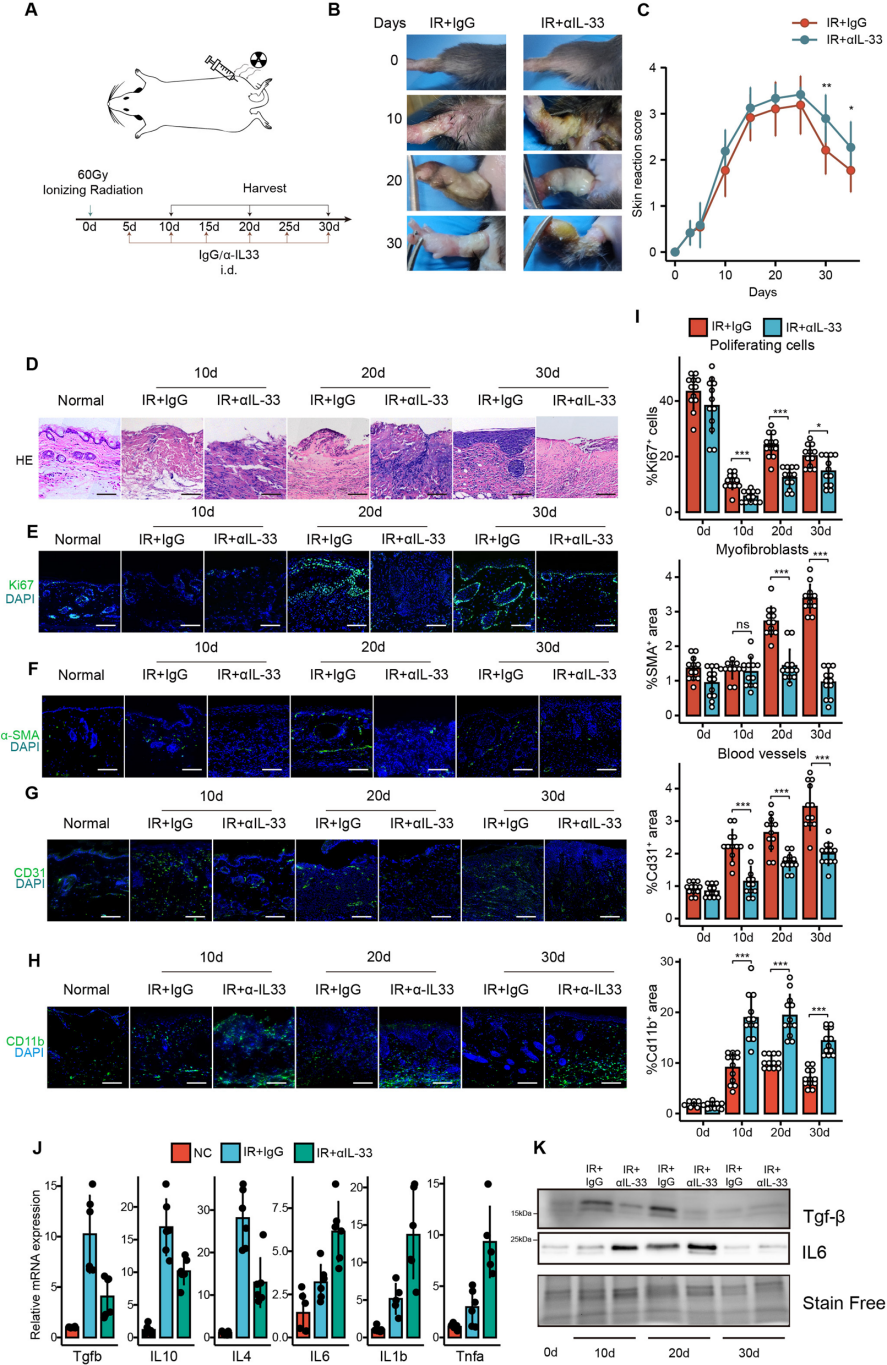
IL-33 improves the healing outcome and modulated the immune microenvironment in RISI. **A** Schematic representation of subcutaneous injection of IL-33 neutralizing antibody or control IgG. **B** Representative images of gross injury after IL-33 neutralizing antibody injection compared to control IgG injection, n = 3. **C** Gross injury scores for the IL-33 neutralizing antibody injection group compared to the control groups, n = 3 for each group. **D** Representative H&E staining images of IL-33 neutralizing antibody injection groups versus control groups. Representative immunofluorescence images of **E** Ki67^+^ proliferating cells, **F** α-SMA^+^ myofibroblasts, **G** CD31^+^ endothelial cells and **H** Cd11b^+^ myeloid immune cells for the IL-33 neutralizing antibody injection groups versus the control groups. **I** Quantitative analysis of Ki67, α-SMA, and CD31 positive signals in the IL-33 neutralizing antibody injection groups versus the control groups. Twelve high-power fields were randomly selected, and positive signal cell ratios (for Ki67) or positive signal areas (for α-SMA and CD31) were analyzed using ImageJ software. **J** qPCR analysis of changes in M2 macrophage marker CD206 and M1 macrophage marker CD86 gene expressions after IL-33 neutralizing antibody injection compared to control groups, n = 6. **K** Western blot indicated a decrease in the anti-inflammatory cytokine Tgf-β expression level and an increase in the pro-inflammatory cytokine IL-6 expression level after IL-33 neutralizing antibody injection, with three repetitions, and representative images was shown. Data were represented as mean ± standard deviation. When variance was met, a *t*-test was used for statistical analysis between two groups, while ANOVA was used for comparisons among three or more groups. If variance wasn’t met, the Mann-Whitney *U* test was used. *p < 0.05, **p < 0.01, ***p < 0.001. Bars represented 100 μm

To assess the importance of IL-33 secreted by senescent fibroblasts in promoting the re-epithelialization and collagen deposition of RISI, we chose to neutralize IL-33 using antibody to investigate its roles. Figure 5A showed a schematic diagram of IL-33 neutralization. As expected, the neutralization of IL-33 resulted in more severe exudation, more prolonged ulceration (Fig. 5B), and higher damage scores (Fig. 5C) in the irradiated tissues compared with the control group. Subsequent pathological analysis of the neutralized tissues revealed reduced epidermal coverage, disorganized dermal tissue structure, and loss of subcutaneous appendages (Fig. 5D). Further immunofluorescence analysis showed reduced cell proliferation after IL-33 neutralization (Fig. 5E, I), decreased fibroblast activation (Fig. 5F, I), and slowed angiogenesis (Fig. 5G, I). These findings were consistent with the inhibitory effect after removal of senescent cells.

IL-33 has been reported as a type 2 cytokine that significantly inhibits the inflammatory response [40], while persistent and widespread inflammation is one of the reasons for difficulty in healing chronic wounds, including RISI. Based on the findings that IL-33 neutralization exacerbated inflammatory cell infiltration in HE staining results, our next step aimed to evaluate the effect of IL-33 on the immune microenvironment in RISI. Immunofluorescence staining for the myeloid immune cell marker Cd11b demonstrated the presence of myeloid immune cell infiltration after irradiation, and IL-33 neutralization exacerbated the inflammatory response (Fig. 5H, I). Next, we performed PCR on various pro- and anti-inflammatory factors, which showed that anti-inflammatory factors, including Tgf-β, IL-10, and IL-4 were significantly down-regulated after IL-33 neutralization. In contrast, the levels of pro-inflammatory factors such as IL-6, IL-1b, and Tnf-α were significantly increased (Fig. 5J). Further, Western blot also demonstrated that IL-33 neutralization led to high expression of the pro-inflammatory factor IL-6, accompanied by a decrease in the pro-repair factor Tgf-β expression level (Fig. 5K). The results suggested that IL-33 secreted by senescent fibroblasts effectively promoted the healing process of RISI wounds and at the same time altered the immune microenvironment of the wounds toward the pro-repair direction.

### The effect induced by IL-33 is upon the polarization state of macrophages

**Fig. 6:**
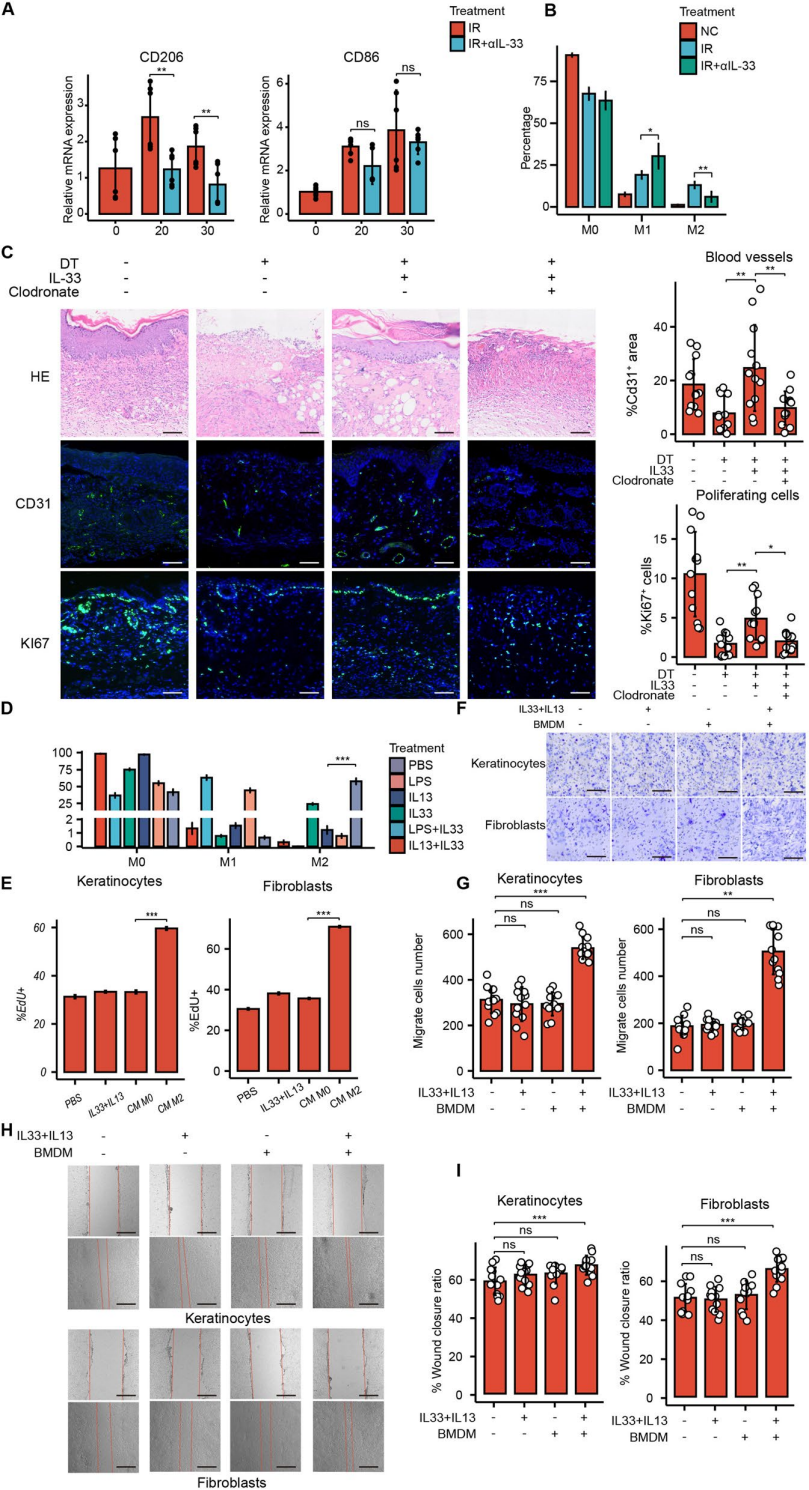
The role of IL-33 is partly dependent on the polarization state of macrophages. **A** qPCR analysis of changes in the gene expression of the M2 macrophage marker CD206 and the M1 macrophage marker CD86 after IL-33 neutralizing antibody injection compared to the control groups (mean ± SD, n = 6). **B** Quantitative analysis of flow cytometry results of the proportion of differently polarized macrophages in skin single-cell suspensions after injection of IL-33-neutralizing antibody compared with controls (mean ± SD, n = 3). **C** Representative H&E staining, CD31 immunofluorescence staining, and Ki67 immunofluorescence images for the following conditions at 20 days post-irradiation: clearance of senescent cells, subcutaneous injection of recombinant IL-33 after clearance of senescent cells, and subcutaneous injection of recombinant IL-33 after clearance of macrophages with Clodronate; n = 3. Quantitative analyses were conducted by randomly selecting 12 high-power fields, and using ImageJ to compute the area with CD31 positive signals and the percentage of Ki67 positive cells. **D** Mouse bone marrow (BM) cells were isolated and cultured in the presence of M-CSF (20 ng/mL). After six days, macrophages were stimulated with LPS (100 ng/mL), IL-13 (10 ng/mL), IL-33 (20 ng/mL), or a combination of these agents for 48 h. Flow cytometry was used to analyze the surface markers CD206 for M2 macrophages and CD86 for M1 macrophages. Quantitative analyses of flow cytometry results for the proportions of M0, M1, and M2 macrophages are presented (mean ± SD, n = 3). **E** Primary fibroblasts or primary keratinocytes were stimulated with PBS, polarization-inducing medium containing IL-33 and IL-13 without macrophages, M0 macrophage-conditioned medium, or conditioned medium from M2 macrophages treated with IL-33 and IL-13. Flow cytometry was used to analyze the percentage of EdU^+^ proliferating cells, with results representing data from three repeated experiments (mean ± SD, n = 3). **F** Transwell assays revealed the macrophage-dependent ability of IL-33 to enhance cell migration. Representative images showing the migratory ability of primary keratinocytes (top) and primary fibroblasts (bottom) incubated under different conditions for 24 h. **G** Quantitative analysis of the number of migrating cells in the Transwell assay of keratinocytes and primary fibroblasts under different stimuli based on 12 randomly selected high magnification fields of view (mean ± SD). **H** Scratch assay demonstrated that IL-33-enhanced cell migration was dependent on macrophages. Representative images showing the migratory ability of primary keratinocytes (top) and primary fibroblasts (bottom) incubated under different conditions for 12 h. The red dashed lines indicated the edge of migrating cells. **I** Quantitative analysis of the wound closure ratio in the scratch assay of keratinocytes and primary fibroblasts under different stimuli based on 12 randomly selected high magnification fields of views (mean ± SD). When variance was met, a *t*-test was used for statistical analysis between two groups, while ANOVA was used for comparisons among three or more groups. If variance was not met, the Mann‒Whitney *U* test was used. *p < 0.05, **p < 0.01, ***p < 0.001. Bars represented 50 μm (**F**, **H**) and 100 μm (**C**). EdU stood for 5-ethynyl-2′-deoxyuridine, and BMDMs for bone marrow-derived macrophages

Immune cells remove tissue debris and microbial contamination from injured tissues while secreting cytokines and growth factors to promote tissue repair and wound closure. However, the immune cell phenotype must be finely regulated to avoid excessive tissue damage. Among them, macrophages are deeply involved in this process, and their transition from a pro-inflammatory to an anti-inflammatory state is critical for tissue repair outcomes. Several studies have demonstrated that ST2, as the sole receptor for IL-33, is widely expressed on the surface of macrophages and that IL-33 effectively promotes macrophage conversion to the M2 phenotype [41–43]. To further investigate whether IL-33 produced by fibroblasts in RISI exerts pro-reepithelialization and collagen deposition effects by altering the polarization state of macrophages, we first conducted a study of the macrophage polarization markers CD206 and CD86 by qPCR analysis (Fig. 6A). Flow cytometry analysis of macrophage polarization markers in tissues similarly confirmed that neutralization of IL-33 significantly inhibited macrophage M2 polarization (Fig. 6B, Additional file 1: Fig. S3). The above experiments demonstrated that IL-33 secreted by senescent fibroblasts could alter macrophage polarization while promoting the healing of RISI.

Next, we further assessed whether the IL-33 pro-repair effect depended on macrophage polarization status alterations. We assessed the effect of IL-33 injection on improving RISI re-epithelialization and collagen deposition after macrophage clearance by Clodronate Liposomes. The successful removal of macrophages by liposomes was shown in Additional file 1: Fig. S4. HE staining showed that removal of senescent cells after irradiation significantly delayed epidermal coverage and ulcer healing, and IL-33 injection partially ameliorated the damage caused by the removal of senescent cells, but scab coverage remained. The repair effect was significantly suppressed by IL-33 injection after macrophage removal. Immunofluorescence staining for the angiogenic indicator CD31 and the proliferation indicator Ki67 similarly showed that IL-33 injection partially ameliorated the decreased levels of vascular infiltration and cellular proliferation caused by senescent cell clearance, but this effect was reversed with macrophage clearance (Fig. 6C). This suggested that the improvement of re-epithelialization and collagen deposition effect exerted by IL-33 partially depending on the presence of macrophages.

To further assess the role of IL-33 in M2 macrophage polarization, we stimulated bone marrow-derived macrophages with IL-33, LPS (an M1 polarization stimulus), IL-13 (an M2 polarization stimulus), or combinations of these cytokines for 48 h. As expected, IL-13 and LPS had specific effects on CD206 and CD86 expression, with IL-13 increasing and LPS decreasing the percentage of CD206^+^ cells and LPS increasing the percentage of CD86^+^ cells. On its own, IL-33 had no significant effect on CD206 and CD86 expression. However, in the presence of IL-13, IL-33 strongly enhanced CD206 expression without affecting CD86 expression (Fig. 6D, Additional file 1: Fig. S5). These results indicated that IL-33 significantly enhances the polarization of mouse M2 macrophages induced by IL-13.

Considering the previously observed promotional effects of IL-33 on collagen deposition and epidermal proliferation, we investigated the effects of IL-33-induced macrophage polarization on the proliferative and migratory capacities of keratinocytes and fibroblasts. Primary mouse keratinocytes and fibroblasts were stimulated for 72 h, and the sources of stimuli were medium from M0 macrophages, medium from IL-33- and IL-13-induced M2 macrophages, and macrophage-free control medium. Flow cytometry analysis showed higher levels of intracellular EdU uptake in M2 macrophage-stimulated keratinocytes and fibroblasts compared with cells stimulated with M0 macrophage or macrophage-free control medium (Fig. 6E, Additional file 1: Fig. S6). Transwell and scratch experiments demonstrated a significant enhancement of keratinocytes and fibroblasts migration after stimulation with M2 macrophage medium (Fig. 6F–I), but the macrophage-free control medium had no significant effect on the migratory capacity of either. These in vitro experiments likewise demonstrated that IL-33 needs to induce M2 macrophage polarization in order to have an effect on keratinocyte and fibroblast migration and proliferation, thereby promoting RISI recovery, rather than acting alone.

## Discussion

As one of the most common side effects of radiotherapy, mild RISI manifesting as desquamation or blistering heals in most cases with routine care such as moisturization, glucocorticosteroids, or silver-ion-containing dressings [44]. However, severe RISI with ulcers would tend to have delayed healing, recurrent breakouts, and severe pain. Unfortunately, the existing generalized treatments for cuts and burns are ineffective. This is mainly because the mechanism of injury caused by ionizing radiation is more specific, and the critical aspects involved in the repair phase of RISI are not yet fully understood, which limits the choice and development of therapeutic approaches. Here, we found that radiation-induced cellular senescence is involved in re-epithelialization and collagen deposition during the repair of RISI. Further, we localized that senescent fibroblasts secreting IL-33 as an SASP that promoted fibroblast activation and epidermal proliferation by affecting macrophage polarization. This result enriches the current understanding of the cell biological events during RISI. It provides a new intervention target for the intervention of RISI, which is expected to be followed by the development of therapeutic tools to accelerate the healing of RISI on this basis.

The most important biological effect of ionizing radiation is the damage to biomolecules, such as DNA, the destruction of which usually leads to cell death or cellular senescence. Norozi et al. found that a very short period after radiation resulted in cell death [45], which was consistent with our observation that large doses of radiation result in massive cell death in the skin in a widespread manner. Lower doses of radiation mainly lead to keratinocyte death, causing wet flaking. Higher doses of radiation lead to dermal cell death and disruption of the structure of the whole skin layer, triggering ulcers [46]. However, the presence of dead cells is rare during collagen deposition or re-epithelialization of the ulcer. At this stage, we observed an accumulation of senescent cells, which was another radiation-induced alteration of the cellular state. Although senescent cells lose their proliferative capacity, they can secrete various cytokines and growth factors, and many studies have also shown that senescent cells play an essential role in the tissue repair process represented by wound healing. Both senescent cell markers and SASP are transiently up-regulated after skin excision in mice and humans, which is critical for wound healing. Demaria et al. [47] found for the first time that senescent fibroblasts and vascular endothelial cells resulting from excision wounds expressed PDGF-AA and that targeted removal of senescent cells followed by supplementation with PDGF-AA reversed the delay in wound healing. In addition, CCN1-induced senescent cells played an aggressive role in pro-heart regeneration, and macrophages expressing p16 and SA-β-Gal had been suggested to be involved in the clearance of dead cells as well as in the transformation of the immune microenvironment [48, 49]. Notably, in chronic wounds, senescent cells may play the opposite role [10, 13, 48, 50–52]. Many studies have shown that senescent cells are detrimental to chronic wound healing. Advanced glycosylation end products in diabetic wounds and hypoxic environments in pressure ulcers similarly lead to cellular senescence, where a proportion of senescent cells that exceeds 15% lead to ulcers with difficulty in healing [53]. The current explanation for the functional heterogeneity of senescent cells is that transient and a few senescent cells are beneficial for tissue repair; however, the presence of a large and persistent number of senescent cells may be counterproductive. Also, there are differences in SASP expression profiles and functions between senescence-inducing conditions and cell types [54]. On this basis, and in conjunction with our experimental results, we hypothesized that the beneficial effects of senescent cells on wound healing in RISI may only exist within a short window of time, and that the prolonged presence of senescent cells may limit tissue repair. Intervention strategies against the emergence of pro-repair senescent cells in RISI may be based on inducing their early production rather than inhibiting their removal.

SASP is a central effector molecule that exerts biological effects in senescent cells; however, the SASP that functions in radiation-induced senescent cells has not been fully revealed. We found that IL-33 was one of the significant SASPs secreted by radiation-induced senescent fibroblasts. IL-33 is a type 2 cytokine with pleiotropic immunomodulatory effects. As a member of the IL-1 family of cytokines, IL-33 is involved in the repair process of a variety of diseases, including diabetic wounds [55] and burn wounds [41]. At the same time, it also exerts an active role in liver regeneration [56]. Previous studies have generally concluded that IL-33 acted as an alarmin, constitutively being expressed in multiple cells such as vascular endothelium and epidermis, and being released to activate the immune response after cellular injury [57]. The activation of the immune response is dependent on the sole receptor for IL-33, ST2, which is highly expressed mainly in ILC2, Th2, and macrophages.ILC2 and Th2, after being activated by IL-33, express type 2 cytokines, mainly IL4 and IL13, which regulate the function of epithelial cells and fibroblasts [42, 58, 59]. As for macrophages, it had been reported that IL-33 accelerates diabetic wound repair by up-regulating the polarization of M2-type macrophages [60]. Wang et al. [61] combined IL-33 with a hydrogel delivery system, which altered the polarization status of macrophages and resulted in superior therapeutic effects in diabetic wound healing. Our results showed that IL-33 enhanced the effect of IL-13-triggered macrophage M2 polarization and that IL-33 administration alone had no significant effect, which was consistent with what was observed by He et al. [60] Since ILC2 and Th2 cells also express ST2 receptors, these two types of immune cells may also be involved in RISI repair as targets of IL-33. Here, we reported for the first time that IL-33 can play an essential role in promoting RISI repair as an SASP secreted by radiation-induced senescent fibroblasts. Recently, the fact that IL-33 could function as a senescent cell SASP was partially elaborated in a hepatocellular carcinoma model, which is consistent with our observations. Yamagishi et al. [62] found that in an obesity-induced hepatocellular carcinoma model, senescent hepatic stellate cells cleave and activate IL-33 by CELA1 protease, and IL-33 in the activated state was released via Gasdermin D into the tumor microenvironment. A similar pattern of IL-33 release was observed in allergic airway epithelium by Kita et al. [63]. However, whether IL-33 release follows the above paradigm in radiation-induced cellular senescence still needs further investigation.

In conclusion, the interactions between senescent cells, IL-33, and macrophages not only highlighted the complexity of RISI repair but also revealed potential targets for therapeutic intervention. First, despite the pro-repairing effects of Senolytics in chronic wounds, caution should be exercised in the use of such drugs in RISI because of the positive role of senescent cells in essential aspects involved in wound repair. Precise regulation of the fate and function of senescent cells according to their characteristics in different periods may play a crucial role in the treatment of RISI. Secondly, based on the pro-repairing effect of IL-33, treatment with this factor applied to the wound in combination with the appropriate delivery material, supplemented by other standard antimicrobial and moisturizing therapies may be feasible.

### Supplementary Information

**Additional file 1:** **Figure S1.** Validation of the p16^DTR−tdTomato^ mice in vivo. **A** qPCR verification of mRNA expression levels of transgenic elements (mean ± SD, n = 3). Immunofluorescence co-localization of primary fibroblast tdTomato autofluorescence with the senescence marker, **B** p16, **C** P21, and **D** SA-β-Gal. **E** CCK-8 assay for cell viability of senescent and control primary dermal fibroblasts at different concentrations of diphtheria toxin (mean ± SD, n = 3). When variance was met, a *t*-test was used for statistical analysis between two groups, while ANOVA was used for comparisons among three or more groups. If variance was not met, the Mann-Whitney *U* test was used. *p < 0.05, **p < 0.01, ***p < 0.001. DTR stood for diphtheria toxin receptor, IR for ionizing radiation, and SA-b-Gal for senescence-associated β galactosidase. **Figure S2.** DNA damage and cellular senescence are persistent after radiation exposure. **Figure S3.** Distribution of senescent cells at different times after irradiation assessed by p16^DTR−tdTomato^ mice. **A** tdTomato autofluorescence in young mice, aged mice, and aged mice after removal of senescent cells using diphtheria toxin. **B** Flow cytometry assessment of the distribution of senescent cells at different times after irradiation. **C** Evaluation of tdTomato autofluorescence and quantitative analysis of senescent cells distribution at different times after irradiation (mean ± SD, n = 3). DT stood for diphtheria toxin. **Figure S4.** Representative data from flow cytometry analysis of the proportions of differently polarized macrophages in skin single-cell suspensions after IL-33 neutralizing antibody injection versus the control groups. **Figure S5.** Representative F4/80 immunohistochemical staining results and their quantitative analysis show adequate clearance of macrophages by Clodronate. Results were expressed as mean ± SD, n = 12. When variance was met, ANOVA was used for comparisons among three or more groups. If variance was not met, the Mann-Whitney *U* test was used. *p < 0.05, **p < 0.01, ***p < 0.001. IR stood for ionizing radiation, and CLO for Clodronate. **Figure S6.** Representative flow cytometry results show BMDM cell polarization under different treatment conditions in vitro. **Figure S7.** Representative flow cytometry results show the effects of different in vitro conditions inducing BMDM on the proliferation of keratinocytes and fibroblasts. **Table S1.** Impairment Gross Performance Rating Scale. **Table S2.** Primers used for qRT-PCR. **Table S3.** Primary antibodies used in this study.

## Data Availability

Data and materials related to this work are available from the corresponding author upon request.
