# Supplementary material for: Senescent fibroblast facilitates re-epithelization and collagen deposition in radiation-induced skin injury through IL-33-mediated macrophage polarization
Source: J Transl Med. 2024 Feb 18;22:176. doi: 10.1186/s12967-024-04972-8 (PMC10874572; doi:10.1186/s12967-024-04972-8)
Supplement: Supplementary file 1 — Additional file 1: Figure S1. Validation of the p16DTR−tdTomato mice in vivo. A qPCR verification of mRNA expression levels of transgenic elements (mean ± SD, n = 3). Immunofluorescence co-localization of primary fibroblast tdTomato autofluorescence with the senescence marker, B p16, C P21, and D SA-β-Gal. E CCK-8 assay for cell viability of senescent and control primary dermal fibroblasts at different concentrations of diphtheria toxin (mean ± SD, n = 3). When variance was met, a t-test was used for statistical analysis between two groups, while ANOVA was used for comparisons among three or more groups. If variance was not met, the Mann-Whitney U test was used. *p < 0.05, **p < 0.01, ***p < 0.001. DTR stood for diphtheria toxin receptor, IR for ionizing radiation, and SA-b-Gal for senescence-associated β galactosidase. Figure S2. DNA damage and cellular senescence are persistent after radiation exposure. Figure S3. Distribution of senescent cells at different times after irradiation assessed by p16DTR−tdTomato mice. A tdTomato autofluorescence in young mice, aged mice, and aged mice after removal of senescent cells using diphtheria toxin. B Flow cytometry assessment of the distribution of senescent cells at different times after irradiation. C Evaluation of tdTomato autofluorescence and quantitative analysis of senescent cells distribution at different times after irradiation (mean ± SD, n = 3). DT stood for diphtheria toxin. Figure S4. Representative data from flow cytometry analysis of the proportions of differently polarized macrophages in skin single-cell suspensions after IL-33 neutralizing antibody injection versus the control groups. Figure S5. Representative F4/80 immunohistochemical staining results and their quantitative analysis show adequate clearance of macrophages by Clodronate. Results were expressed as mean ± SD, n = 12. When variance was met, ANOVA was used for comparisons among three or more groups. If variance was not met, the Mann-Whitney U test [file 12967_2024_4972_MOESM1_ESM.docx]

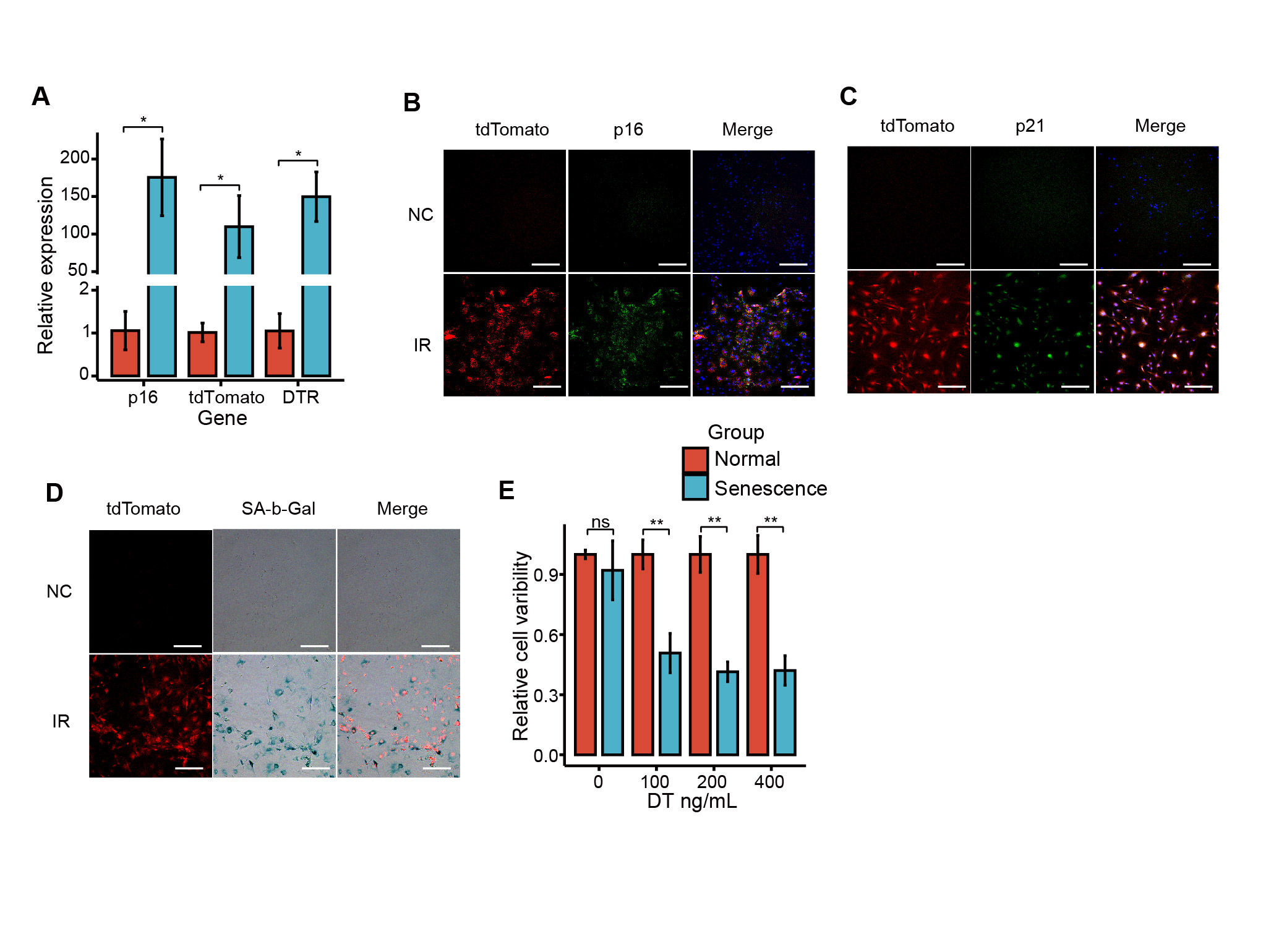


## Figure S1. Validation of the p16^DTR-tdTomato^ mice in vivo.

(A) qPCR verification of mRNA expression levels of transgenic elements (mean±SD, n = 3). Immunofluorescence co-localization of primary fibroblast tdTomato autofluorescence with the senescence marker (B) p16, (C) P21, and (D) SA-β-Gal. (E) CCK-8 assay for cell viability of senescent and control primary dermal fibroblasts at different concentrations of diphtheria toxin (mean±SD, n = 3). When variance was met, a t-test was used for statistical analysis between two groups, while ANOVA was used for comparisons among three or more groups. If variance was not met, the Mann-Whitney U test was used. *p < 0.05, **p < 0.01, ***p < 0.001. DTR stood for diphtheria toxin receptor, IR for ionizing radiation, and SA-b-Gal for senescence-associated β galactosidase.


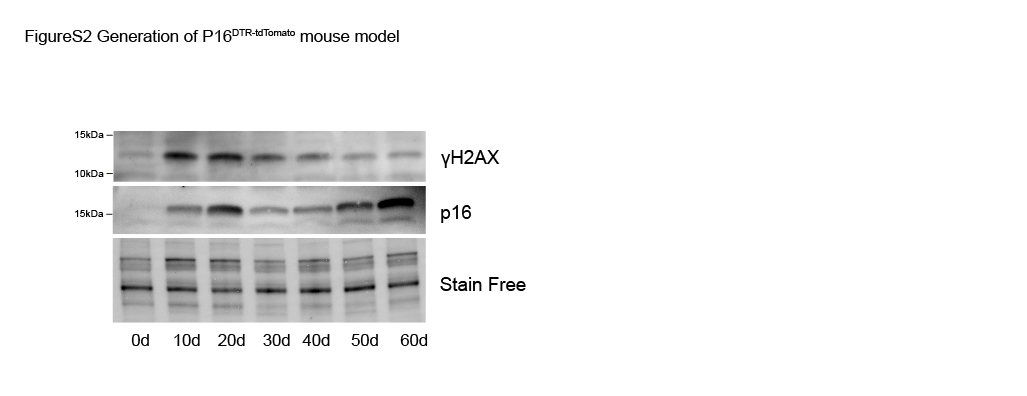


## Figure S2. DNA damage and cellular senescence are persistent after radiation exposure.


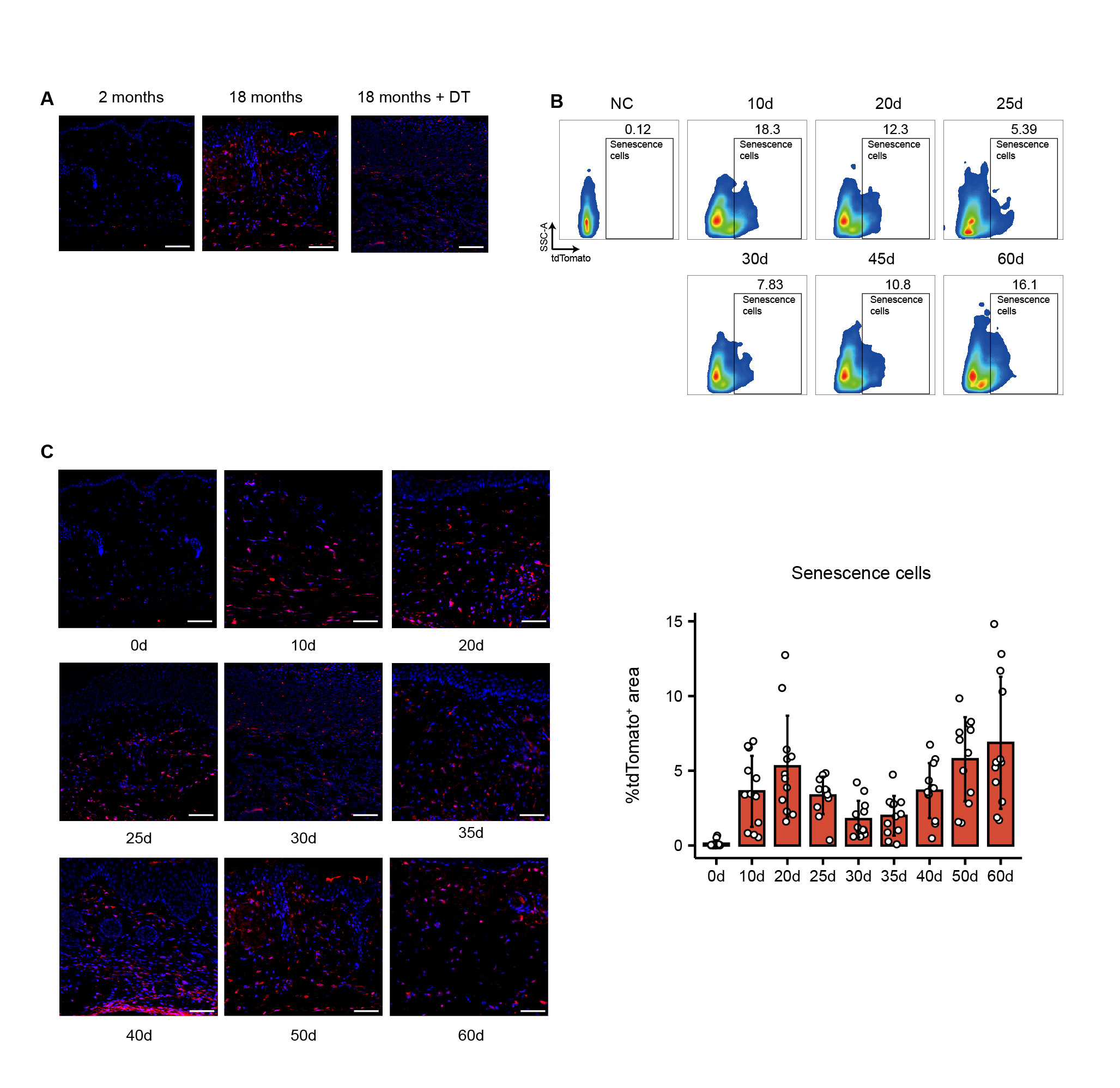


## Figure S3. Distribution of senescent cells at different times after irradiation assessed by p16^DTR-tdTomato^ mice.

(A) tdTomato autofluorescence in young mice, aged mice, and aged mice after removal of senescent cells using diphtheria toxin. (B) Flow cytometry assessment of the distribution of senescent cells at different times after irradiation. (C) Evaluation of tdTomato autofluorescence and quantitative analysis of senescent cells distribution at different times after irradiation (mean±SD, n = 3). DT stood for diphtheria toxin.


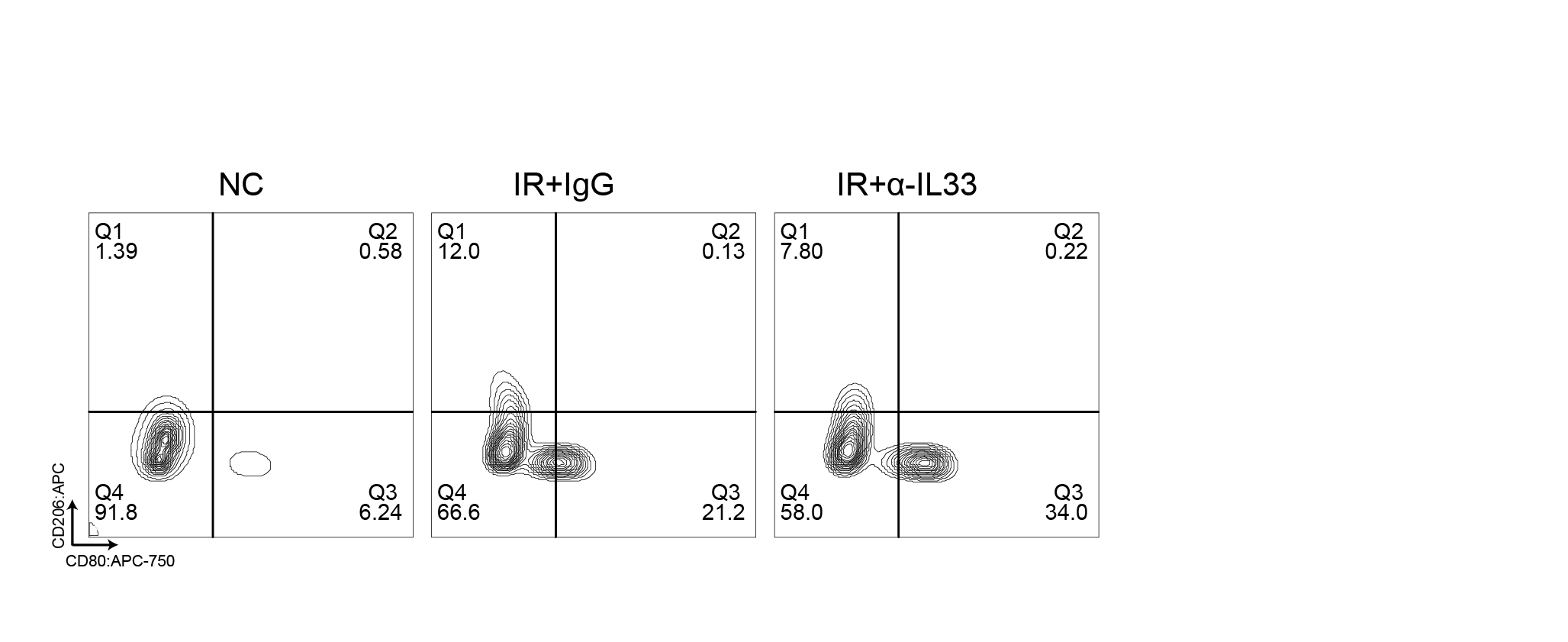


## Figure S4. Representative data from flow cytometry analysis of the proportions of differently polarized macrophages in skin single-cell suspensions after IL-33 neutralizing antibody injection versus the control groups.


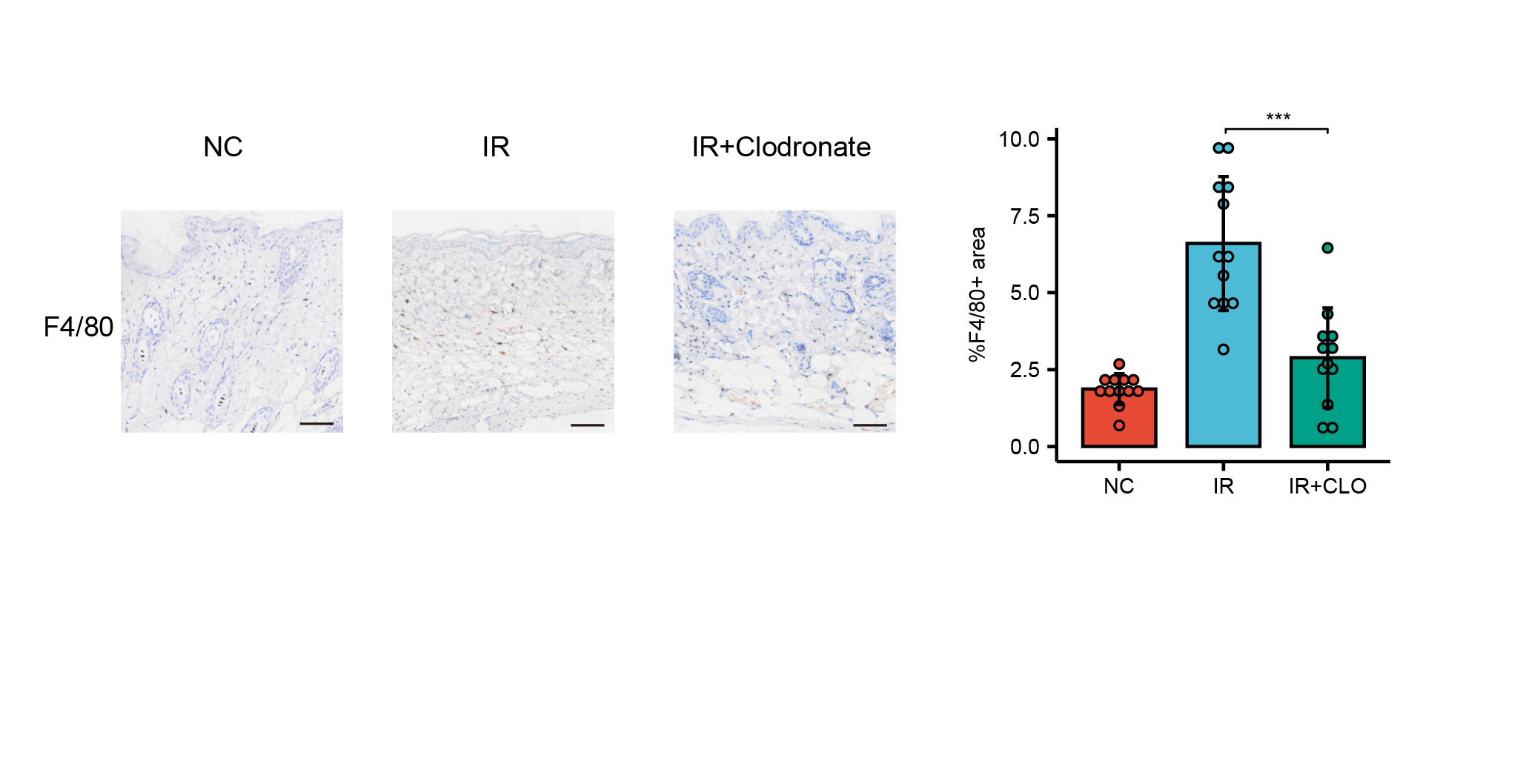


## Figure S5. Representative F4/80 immunohistochemical staining results and their quantitative analysis show adequate clearance of macrophages by Clodronate.

Results were expressed as mean ± SD, n = 12. When variance was met, ANOVA was used for comparisons among three or more groups. If variance was not met, the Mann-Whitney U test was used. *p < 0.05, **p < 0.01, ***p < 0.001. IR stood for ionizing radiation, and CLO for Clodronate.


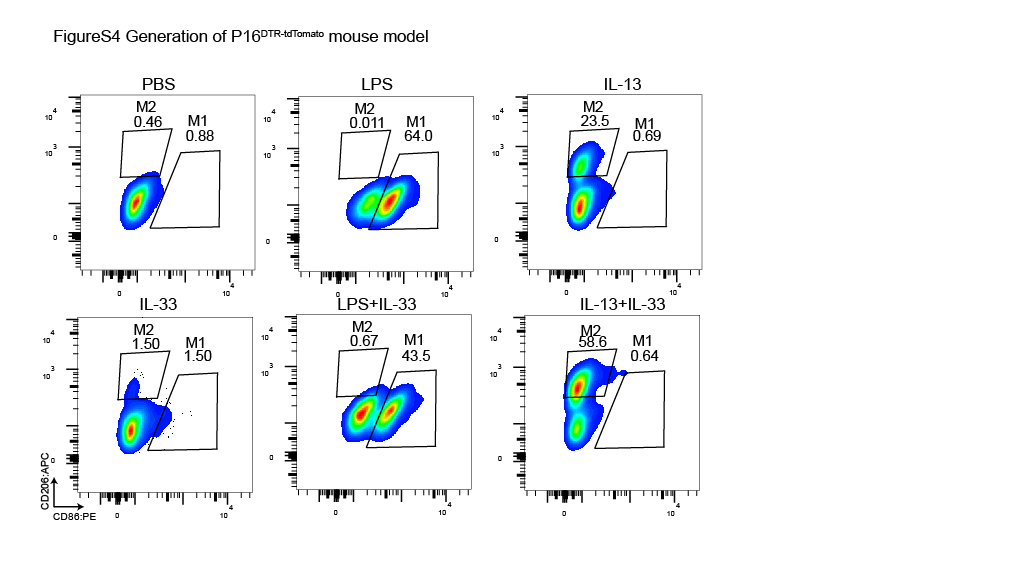


## Figure S6. Representative flow cytometry results show BMDM cell polarization under different treatment conditions in vitro.


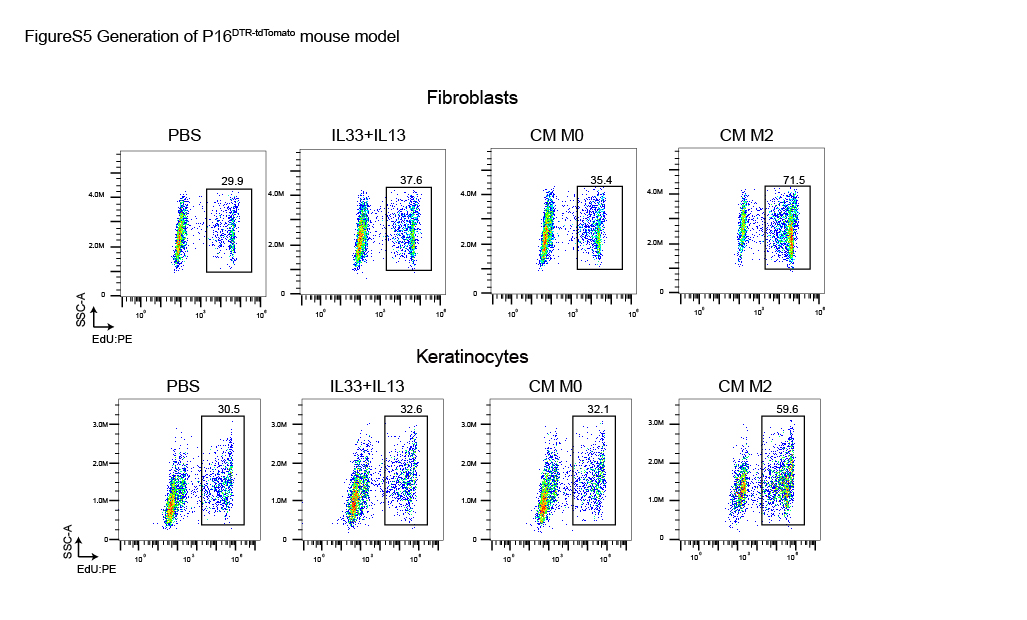


## Figure S7. Representative flow cytometry results show the effects of different in vitro conditions inducing BMDM on the proliferation of keratinocytes and fibroblasts.

## Table S1: Impairment Gross Performance Rating Scale

| Score | Observation |
| --- | --- |
| 0.5 | 50/50 doubtful if there is any difference from normal |
| 1- | Definite but slight abnormality |
| 1 | Definite abnormality with reddening |
| 1+ | Severe reddening and/or white scales and/or puffiness |
| 1.5 | Moist breakdown in one very small area, with scaly or crusty appearance |
| 1.5+ | Moist desquamation in small areas (more definite than 1.5) |
| 2 | Breakdown of large area, possibly moist in places |
| 2.5 | Breakdown of large areas of skin with definite moist exudate |
| 3 | Breakdown of most of skin with moist exudate |
| 3.5 | Complete moist breakdown of limb - often stuck to body |

## Table S2: Primers used for qRT-PCR

| Genes | Forward | Reverse |
| --- | --- | --- |
| Cd206 | ATGGATTGCCCTGAACAGCA | TGTACCGCACCCTCCATCTA |
| Cd86 | CTTACGGAAGCACCCACGAT | CGGCAGATATGCAGTCCCAT |
| Cdkn2a | GTGGGCATTTCTTGCGA | TTTGGTTCTGCCATTTGCT |
| Dtr | ATGGTGAGCAAGGGCGAGGA | ACAGGATCTGTCCCAGGCGA |
| tdTomato | CCACAAGCACTGGCCACACCAAGCA | TGAGAAGCCCCACGATGACCAGCA |
| Cdkn2a | CTTCGCCGAGCAGTTTCGT | TCAATCCCATCAGCCATTTCC |
| Igf1 | GTGGGGGCTCGTGTTTCTC | GATCACCGTGCAGTTTTCCA |
| Col3a1 | CTGTAACATGGAAACTGGGGAAA | CCATAGCTGAACTGAAAACCACC |
| Col1a1 | GCTCCTCTTAGGGGCCACT | CCACGTCTCACCATTGGGG |
| Ccn1 | CGCCAACACCTCTACTCTCC | TCCACCTTGGTAACATGCTGA |
| Postn | CCTGCCCTTATATGCTCTGCT | AAACATGGTCAATAGGCATCACT |
| β-actin | CTAAGGCCAACCGTGAAAAG | ACCAGAGGCATACAGGGACA |

## Table S3: Primary antibodies used in this study

| Target | Company | Catalogue number |
| --- | --- | --- |
| ki-67 | Abcam | ab15580 |
| α-SMA | Millipore | A2547 |
| CD31 | Abcam | ab28364 |
| p16 | Abcam | ab54210 |
| tdTomato | Oasis | OB-RB013-02 |
| CD45 | Biolegend | 157214 |
| CD26 | eBioscience | 45-0261-82 |
| CD44 | eBioscience | 17-0441-82 |
| F4/80 | Biolegend | 123131 |
| CD31 | eBioscience | 63-0311-82 |
| Tgf-β | Abcam | ab215715 |
| IL-6 | Abcam | ab9324 |
| γ-H2AX | Abcam | ab81299 |
| p16 | Santa Cruz | sc1661 |
